# Implications of Below-Ground Allelopathic Interactions of *Camelina sativa* and Microorganisms for Phosphate Availability and Habitat Maintenance

**DOI:** 10.3390/plants12152815

**Published:** 2023-07-29

**Authors:** Diana Hofmann, Björn Thiele, Meike Siebers, Mehdi Rahmati, Vadim Schütz, Seungwoo Jeong, Jiaxin Cui, Laurent Bigler, Federico Held, Bei Wu, Nikolina Babic, Filip Kovacic, Joachim Hamacher, Georg Hölzl, Peter Dörmann, Margot Schulz

**Affiliations:** 1 IBG-3: Agrosphäre, Forschungszentrum Jülich GmbH, 52428 Jülich, Germany; d.hofmann@fz-juelich.de (D.H.); b.thiele@fz-juelich.de (B.T.); m.rahmati@fz-juelich.de (M.R.); b.wu@fz-juelich.de (B.W.); 2 IMBIO Institute of Molecular Physiology and Biotechnology of Plants, University of Bonn, 53115 Bonn, Germanyv_schuetz@snu.ac.kr (V.S.); hoelzl@uni-bonn.de (G.H.); doermann@uni-bonn.de (P.D.); 3 Department of Soil Science and Engineering, University of Maragheh, Maragheh 83111-55181, Iran; 4 Department of Chemistry, University of Zurich, CH-8057 Zurich, Switzerland; laurent.bigler@chem.uzh.ch (L.B.); federico.held@uzh.ch (F.H.); 5 Institute of Molecular Enzyme Technology, Heinrich-Heine-University of Düsseldorf and Forschungszentrum Jülich GmbH, 52428 Jülich, Germanyf.kovacic@fz-juelich.de (F.K.); 6 Plant Diseases and Crop Protection, Institute of Crop Science and Resource Conservation, University of Bonn, 53115 Bonn, Germany; hamacher@uni-bonn.de

**Keywords:** allelopathy, apatite, phosphate solubilization, *Camelina sativa*, glucosinolates, microorganisms, *Penicillium aurantiogriseum*, cyclo(L-Leu-L-Pro)

## Abstract

Toxic breakdown products of young *Camelina sativa* (L.) Crantz, glucosinolates can eliminate microorganisms in the soil. Since microorganisms are essential for phosphate cycling, only insensitive microorganisms with phosphate-solubilizing activity can improve *C. sativa’s* phosphate supply. In this study, ^33^P-labeled phosphate, inductively coupled plasma mass spectrometry and pot experiments unveiled that not only *Trichoderma viride* and *Pseudomonas laurentiana* used as phosphate-solubilizing inoculants, but also intrinsic soil microorganisms, including *Penicillium aurantiogriseum*, and the assemblies of root-colonizing microorganisms solubilized as well phosphate from apatite, trigger off competitive behavior between the organisms. Driving factors in the competitiveness are plant and microbial secondary metabolites, while glucosinolates of *Camelina* and their breakdown products are regarded as key compounds that inhibit the pathogen *P. aurantiogriseum*, but also seem to impede root colonization of *T. viride*. On the other hand, fungal diketopiperazine combined with glucosinolates is fatal to *Camelina*. The results may contribute to explain the contradictory effects of phosphate-solubilizing microorganisms when used as biofertilizers. Further studies will elucidate impacts of released secondary metabolites on coexisting microorganisms and plants under different environmental conditions.

## 1. Introduction

*Brassica* mulches are used for biofumigation to reduce weed growth and crop diseases [1,2]. Plant species commonly used for biofumigation are cabbage, Indian mustard, rapeseed, white mustard, and oil radish. The biocidal activity of *Brassica* plants originates from the hydrolysis of their characteristic secondary metabolites with high allelopathic potential, the glucosinolates, resulting in the release of several breakdown products such as toxic isothiocyanates (ITCs). Another Brassicacea, *Camelina sativa* (L.) Crantz, was recently recognized as a plant probably suitable for biofumigation [3,4]. *Camelina sativa*, grown under nutrient sufficient conditions, contains three main aliphatic glucosinolates: glucoarabin (9-(methylsulfinyl) nonyl-glucosinolate, GS9), glucocamelinin (10-(methylsulfinyl) decyl-glucosinolate, GS10), and 11-(methylsulfinyl) undecyl-glucosinolate (GS11), [5]. The corresponding isothiocyanate derivatives are 9-(methylsulfinyl) nonyl-ITC (9-MSITC, arabin), 10-(methylsulfinyl) decyl-ITC (10-MSITC, camelinin) and 11-(methylsulfinyl) undecyl-ITC (11-MSITC). The antimicrobial activity of arabin was previously demonstrated [6]. However, the glucosinolate content in *Camelina* shoots decreases with seedling growth. While 3-day-old seedlings contain about 60% of the glucosinolates originally present in the seeds, only up to 25% are left in 7-day-old seedlings. Mature *Camelina* plants exhibit an organ-specific accumulation of the glucosinolates with high contents in the roots and seeds, while the shoots are almost free of glucosinolates [7,8].

Profuse studies have evidenced that glucosinolate breakdown products have negative impacts on the soil microbial community structure [9]. Hansen et al. [10] report reduced fungal and mycorrhizal abundance and lower grain yield with spring wheat when *Brassica napus* was used as a rotational crop. In another cropping systems experiment with winter wheat and *C. sativa*, Hansen et al. [4] demonstrated a temporary decline in the total microbial biomass encompassing fungi, mycorrhizae, Gram positive and negative bacteria.

The biosynthesis of glucosinolates in *Brassica* plants adapts rapidly to changing nutrient conditions. Sulfur and nitrogen deficiency reduce the biosynthesis, while phosphate (PO_4_^3−^) limitation affects aliphatic glucosinolate biosynthesis positively or negatively [11,12]. The glucosinolate content in *C. sativa*, for instance, depends on N and S availability [13]. P deficiency increased the accumulation of specific glucosinolates in *Arabidopsis* [14], while Frerigmann et al. reported a decrease of indole glucosinolates [15].

Microorganisms are essential for phosphorus (P)—cycling, encompassing the lithosphere, hydrosphere and biosphere. Many of the microorganisms, including those that are isothiocyanate (ITC) sensitive and insensitive, can solubilize phosphate, e.g., from inorganic resources such as apatite. Nevertheless, phosphate deficiency affects many soil- and plant-associated microorganisms. Suffering from P limitation reduces their growth and reproduction. Attempts at compensation demand metabolic energy inputs, for instance for phosphate solubilization. In consequence, changes in microbial biodiversity, species interactions and impacts on associated plants must be expected. Oliverio et al. [16] point out that P limitations are of disadvantage for copiotroph microorganisms but favor the abundance of oligotroph microorganisms in the soil; thus, microbial communities are modulated. Under these conditions, oligotroph microorganisms can compete with plants for phosphate.

P limitation influences not only the biosynthesis of glucosinolates but also of other secondary metabolites in many species. As an example, the biosynthesis of anthocyanins is enhanced under P conditions [17,18]. Low phosphate conditions change the secondary metabolite biosynthesis in bacteria [19]. The influence of nutrients on fungal secondary metabolite production is well known [20]. The negative impact of allelopathic secondary metabolites on nutrient uptake of receiver plants and the often-described enhanced release of allelochemicals by donor plants under phosphate deficiency affects the biodiversity of ecosystems [21,22]. Chevrette et al. [23] conclude from their studies with a model microbial community that secondary metabolites are crucial for shaping interaction networks.

Presumably, P deficiency changes the allelopathic behavior of fungi and may adapt the release of biocidal molecule cocktails for suppression of plant and microbial competitors. On the other hand, breakdown products of glucosinolates are inhibitory for many fungal species. Fungicidal isothiocyanates may be differentially abundant under phosphate deprivation, which changes the allelopathic potential, their interactions with microorganisms and biofumigant properties of *Brassica* species.

The loss of microbial fitness due to nutrient deficiencies combined with plant- or microbial-derived allelochemicals and the resulting impacts on plants are seldom considered in agriculture. This study addresses mutual interferences between *Camelina* and microorganisms under P-deficient conditions. In the first part, we direct microbial capacities of phosphate solubilization from apatite and uptake of the solubilized phosphate by *C. sativa* when grown in a P-depleted soil. In the second part, the glucosinolate break down by microorganisms or myrosinase and the influence of resulting products on fungal growth are studied. Focusing on a dominant soil fungus existing in the P-depleted soil, we addressed features of the entangled allelochemical- and phosphate-driven interferences between *Camelina*, the fungus, root-associated and added microorganisms. We also show in the third part that shoot material from mature *Camelina* plants almost lacking glucosinolates has no apparent fungicidal properties, yet nevertheless modulates the soil microbial diversity in a soil rich in organic matter. We hypothesize an important role of allelopathic secondary metabolites in the phosphate competitiveness of plants and microorganisms, commonly underestimated in agriculture.

## 2. Results

### 2.1. Rhizotron Experiments—Uptake of Phosphate Solubilized from ^33^P-Apatite

The uptake of solubilized ^33^P-labeled phosphate [24,25] by *C. sativa* was studied in the presence and absence of the inoculants *Trichoderma viride* and *Pseudomonas laurentiana*, both applied as suspensions to the root tips.

**Figure 1:**
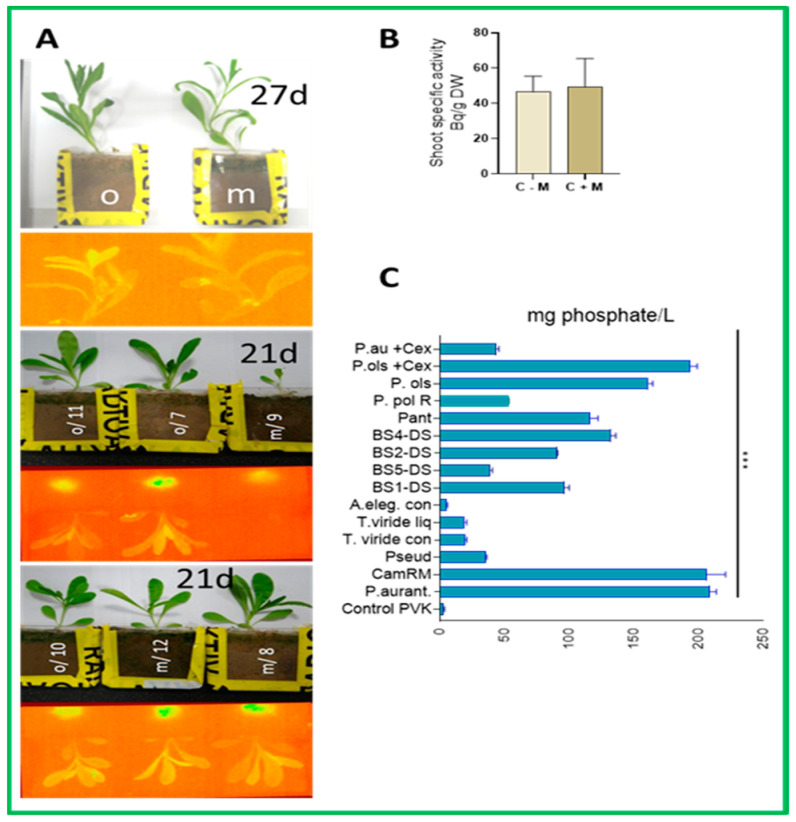
(**A**) Representative uptake experiment of radiolabeled P from ^33^P-apatite by *C. sativa* seedlings after 21 and 27 days: m, in the presence of *T. viride* and *P. laurentiana* inoculants; o, without inoculants, both given with the number of the rhizotron. The inoculations had no clear influence on phosphate uptake (*n* = 6 per plant species). (**B**) Uptake of ^33^P (specific activity Bq/g dry weight,) from solubilized ^33^P-apatite. C-M, C+M: *Camelina* seedlings without *T. viride* and *P. laurentiana* (−) and inoculated (+). Differences in ^33^P uptake between inoculated and non-inoculated samples were not significant. *n* = 3/species and treatment (*t*-test). (**C**) Capacity of phosphate solubilization from apatite by different microorganisms and microbial consortia. Free phosphate was measured by ICP-MS analyses of PVK medium and the media after culturing the microorganisms. *n* = 3 with three technical replicates; significance compared to the control ***: *p* ≤ 0.001 (*t*-test). See also Figure S1. P. au + Cex: *P. aurantiogriseum* + *Camelina* extract added; P. ols + Cex: *P. olsonii* + *Camelina* extract added; P.ols: *P. olsonii* from *Camelina* seeds; P. pol R: *Paenibacillus polymyxa* from *Camelina* roots; Pant: *Pantoea ananatis* isolate from *A. elegans*; *Pseudomonas spec.* (BS4-DS); *Rhodococcus* spec. (BS2-DS); *Arthrobacter spec.* (BS5-DS); *Paenibacillus spec.* (BS1-DS); Ac. eleg. con: *Actinomucor elegans* consortium; *T. viride*: liq. *T. viride* grown in liquid culture; T. viride con. *T. viride* consortium; Pseud: *P. laurentiana*; CamRM: *Camelina* root surface microorganisms; P. aurant: *P. aurantiogriseum*; Control PVK: Control PVK medium.

^33^P–imaging revealed first radioactivity in the leaves at 8 to 9 days after planting. Radioactive ^33^P incorporation increased continuously until the shoots were harvested after 27 days, indicating microbial solubilization of ^33^P from the apatite (Figure 1A). The development of many *Camelina* seedlings was retarded in the presence of *T. viride* and *P. laurentiana* during early growth, and other seedlings died a few days after planting. However, most of the surviving *Camelina* plants caught up with the early growth retardation until harvest. Quantification of absorbed ^33^P indicated that inoculation with *T. viride* and *P. laurentiana* did not enhance phosphate uptake compared with equally developed control plants (Figure 1B).

Any attempt to reisolate *T. viride* from the roots, collected from the rhizotron soil after the radioactivity has subsided, failed. It is therefore likely that the fungus did not establish an association with the plants in the rhizotron experiment. The most viable and dominant bacteria from inoculated roots, which could be cultured on Sabouraud agar, were *Pseudomonas* species, among them probably *P. putida*, while *Acinetobacter spec.* were identified from the non-inoculated roots. Presently, further studies are necessary to verify if *P. laurentiana* is among the colonizing *Pseudomonas* species.

### 2.2. Pot Experiment—Growth of Camelina Seedlings on Dikopshof Soil

These results pointed to other active phosphate-solubilizing microorganisms in the Dikopshof soil that render inoculations of the root tips with *T. viride* and *P. laurentiana* suspensions ineffective and perhaps make microorganisms able to kill *Camelina* seedlings. Also, phosphate-solubilizing microorganisms that have already colonized the roots may be in competitive interaction with the inoculants and soil microorganisms. Therefore, pot experiments using nonsterilized and sterilized Dikopshof soil supplemented with unlabeled apatite were performed. The experiment was started with 3-day-old seedlings of *Camelina* containing still high amounts of glucosinolates.

**Figure 2:**
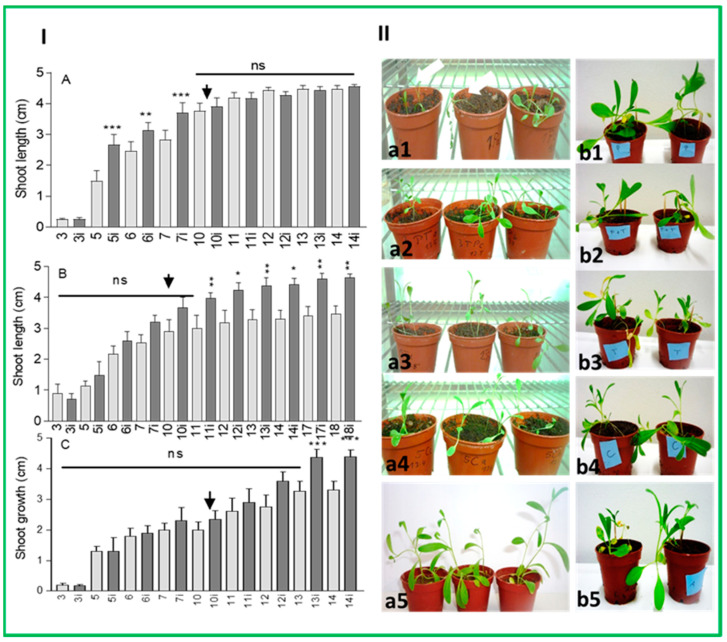
(**I**) Pot experiment with nonsterile and sterilized apatite containing Dikopshof soil and seedlings of *Camelina*. X axis: age (days) of non-inoculated seedlings (gray bars) and inoculated ones (i/dark grey bars). The arrows: P-free fertilizer application. *Camelina*: (**A**) nonsterile soil + *T. viride*, *P. laurentiana*; (**B**) sterilized soil + *T. viride*, *P. laurentiana*; (**C**) sterilized soil + *A. elegans* consortium. T-test; * *p* < 0.05; ** *p* < 0.001; *** *p* < 0.001; ns not significant. (**II**) Pot experiments with sterilized 0-soil supplemented with mycelia of *P. aurantiogriseum* (**a1**,**b1**); *P. aurantiogriseum* + the *T. viride* consortium (**a2**,**b2**); *T. viride* consortium alone (**a3**,**b3**); controls without fungi (**a4**,**b4**); inoculated with *A. elegans* consortium (**a5**,**b5**). a: *Camelina* in 0-soil + apatite; b, *Camelina* + P containing fertilizer; Arrows: dying plants.

The growth of surviving *Camelina* seedlings in nonsterilized soil was first significantly stimulated in the presence of *T. viride* and *P. laurentiana*, evidencing a contribution of the inoculants to plant growth promotion. Later, the difference between inoculated and non-inoculated seedlings was abolished (Figure 2IA). With sterilized soil, inoculation led again to a stimulatory but not significant effect between day 3 and 7 after planting (Figure 2IB). The stimulation turned significant between day 10 and 15. Shoot growth of the non-inoculated *Camelina* plants was retarded in the sterilized soil (Figure 2IB). The results provide clues that soil microorganisms may contribute to phosphate solubilization. Their elimination by sterilization reduces the amount of solubilized phosphate. To address the question whether an *Actinomucor elegans* consortium possessing plant protecting properties can also promote *Camelina*, this consortium was applied to the plants [26] 11. Indeed, the *A. elegans* consortium significantly stimulated the growth of *Camelina* after day 13 (Figure 2IC) in sterilized soil. Taken together, growth monitoring disclosed the existence of interfering organisms, which were eliminated by sterilization.

### 2.3. Abundant Microorganisms in the Dikopshof Soil, on Roots Surfaces of Agar-Grown Camelina and on the Seed Coat

**Scheme 1:**
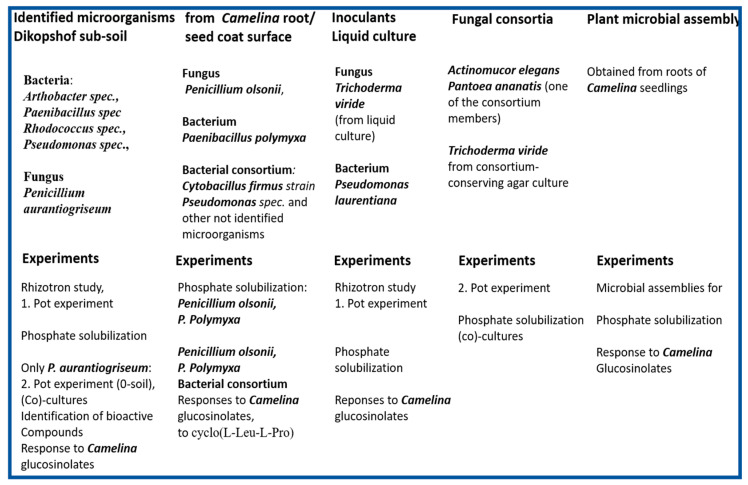
Aforementioned and named after microorganisms addressed in this study. Liquid culture of *T. viride* does not conserve the consortium.

Microbial plating experiments revealed that the Dikopshof soil harbored only a few microorganisms cultivable on Sabouraud agar, among them a fungus that overgrew other soil microorganisms during progressing cultures (Figure S4). The fungus was identified as *Penicillium aurantiogriseum*, a plant pathogen known to release numerous phytotoxic and antimicrobial secondary metabolites [27,28]. *P. aurantiogriseum* developed less mycelium when grown in PVK liquid medium, compared to Sabouraud medium (Figure S2 and Figure 1A,C). Some of the soil bacteria were identified as belonging to the genera *Paenibacillus*, *Rhodococcus*, *Arthrobacter* and *Pseudomonas*. Also, some of the microorganisms colonizing the *Camelina* root surface and seed coat, when seedlings were cultured on Pikovskaya (PVK) agar, were analyzed. *Penicillium olsoni* and *Paenibacillus polymyxa* were found on *Camelina* seed coats and seedlings (Figure S2). On PVK, most roots were heavily covered with yellow colonies composed of several species (see below), among them a *Cytobacillus firmus* strain and a yet unidentified *Pseudomonas* species. The yellow bacterial consortium was not further investigated because it degraded over time. The results underline the variability of root-colonizing microorganisms, depending on the growth conditions. Scheme 1 gives an overview of the microorganisms present in the Dikopshof soil, and further microorganisms used for experiments in this study, here and later in the text.

### 2.4. Coculture of Camelina Seedlings with Fungi in 0-Soil Pot Experiments

To study the interaction of the two fungal consortia (*T. viride*, *A. elegans*) for supporting plant growth, older seedlings of *Camelina* were grown in sterilized 0-soil, a nutrient-deficient artificially mixed substrate supplemented with apatite. Seeds were directly germinated in the pots and after 7 days, when the *Camelina* seedlings had lost about 75% of their glucosinolates, agar plugs covered with mycelia of *P. aurantiogriseum*, the *T. viride* or the *A. elegans* consortium, respectively, were placed into the soil. In the arrangements with only *P. aurantiogriseum*, 60–70% of *Camelina* plants died (Figure 2II). When plugs of the *T. viride* consortium were added to *P. aurantiogriseum*, the development of *Camelina* was comparable to the controls (no fungus) and to plants in pots containing only plugs with the *T. viride* consortium. These results indicate that *P. aurantiogriseum* is toxic to *Camelina* in sterilized 0-soil, and this toxicity can be overcome by addition of the *T. viride* consortium. Addition of plugs with the *A. elegans* consortium was stimulatory to the growth of *Camelina* seedlings, as already observed in the first pot experiment. Interestingly, the *A. elegans* consortium overgrew *P. aurantiogriseum* when cocultured on agar (Figure S2(4d)). The growth behavior of *Camelina* in the presence of the fungi was compared to growth on sterilized 0-soil supplied with phosphate containing fertilizer. Phosphate supply resulted in a different growth behavior of *Camelina* in the presence of *P. aurantiogriseum*, as the fungus did not inhibit *Camelina*, indicating that the negative effect of *P. aurantiogriseum* on *Camelina* seedlings occurs only under phosphate deficiency conditions.

### 2.5. Evaluation of the Microbial Capacity for Phosphate Solubilization

Microorganisms mentioned in Scheme 1 were evaluated for their ability of phosphate solubilization by measuring the phosphate released from apatite using inductively coupled plasma mass spectrometry (ICP-MS). The Dikopshof soil microorganisms *Pseudomonas spec.*, *Rhodococcus spec.*, *Arthobacter spec.*, *Paenibacillus spec*., the total washable microbial collection from root surfaces of *Camelina* grown on cheesecloth, single organisms from *Camelina* seed coat/root (*P. olsonii, P. polymyxa)*, *P. aurantiogriseum*, the *T. viride* suspension, the *T. viride* consortium, the *P. laurentiana* suspension, the *A. elegans* consortium and its consortium member *P. ananatis* were incubated with apatite in PVK medium, and their ability to release phosphate was quantified by ICP-MS.

*P. aurantiogriseum* displayed a high capacity to solubilize phosphate (Figure 1C). The microbial assembly collected from *Camelina* root surfaces had a similar capacity. The *T. viride* consortium and *T. viride* from liquid culture reached only 9–11%, *P. laurentiana* about 20% of the amount of phosphate solubilized by *P. aurantiogriseum*. With the *A. elegans* consortium, almost no free phosphate was measured, but phosphate could be trapped within the nexus of the cotton-like mycelium. This explanation is supported by the relatively high phosphate solubilization found with the *P. ananatis* isolate from the *A. elegans* consortium (Figure 1C). The evaluation of phosphate solubilization confirmed the presence of phosphate-solubilizing microorganisms in the Dikopshof soil, the presence of P-solubilizing microorganisms associated with the root surfaces and with the seeds of *Camelina*. The phosphate solubilization activity of the root-colonizing microbial assemblies, the fungi *P. aurantiogriseum* and *P. olsonii* and further microorganisms could be visualized by culturing on PVK agar plates (Figure S1). The unstable, yellow bacterial consortium-colonizing *Camelina* roots could not be used for the measurement of phosphate solubilization by ICP-MS, as culturing of the intact consortium was not possible in liquid PVK for three days. This consortium exhibited the strongest P-solubilization on PVK agar plates (see below).

### 2.6. Camelina´s Antifungal Weapon: Glucosinolate Breakdown by Soil Bacteria

**Figure 3:**
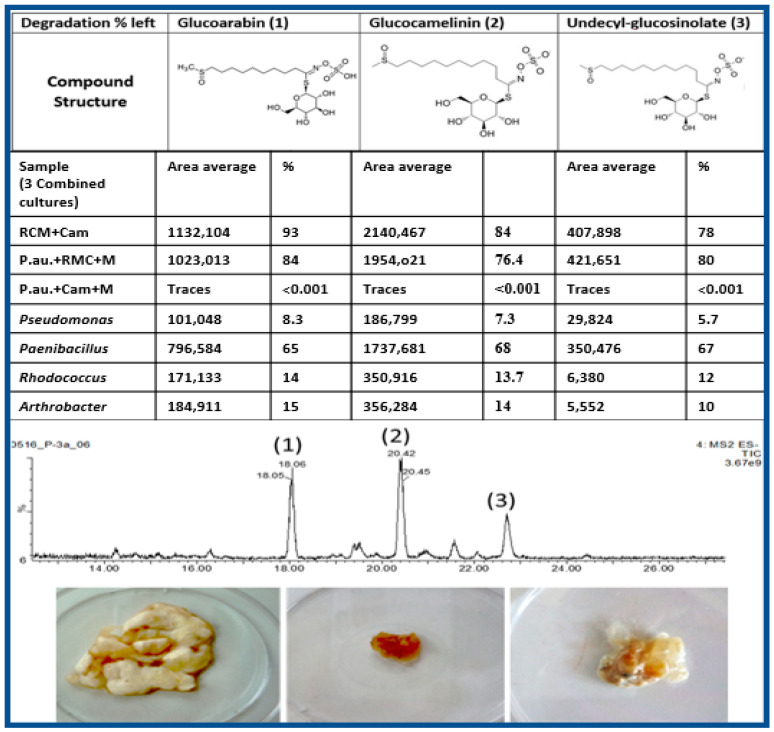
Capacity of different microorganisms to break down glucosinolates and inhibitory effects on *P. aurantiogriseum*. UHPLC-MS analyses of culture media (PKV) supplemented with *Camelina* extract (Cam), (methanolic, re-dissolved in water). Culture media (PKV) supplemented with *Camelina* extract (Cam) were incubated with different microorganisms and glucosinolates measured by UHPLC-MS. P. au.: *P. aurantiogriseum*; RCM: *Camelina* root associated microorganisms; M: myrosinase; *Pseudomonas, Paenibacillus, Rhodococcus*, *Arthrobacter* species are isolates from Dikopshof soil. Triplicates of the different culture supernatants were combined to one sample, technical replicates: *n* = 3. The amounts of the three glucosinolates left after three days of culturing is given in % of the control (no microorganisms added; 100%, calculated from peak areas). The characteristic chromatogram shows glucoarabin (GS9), glucocamelinin (GS10) and undecyl-glucosinolate (GS11). The picture below the chromatogram illustrates the strongly inhibited growth of *P. aurantiogriseum* when incubated in liquid PKV medium with *Sinapis alba* myrosinase added to the methanolic *Camelina* extract redissolved in water (middle) and incubation in presence of myrosinase containing aqueous extract of *Camelina* seedlings (right) in comparison to the control (methanolic *Camelina* extract redissolved in water without myrosinase, left), (*n* = 6).

The results of the *Camelina* growth studies under phosphate deficiency and of the phosphate-solubilizing microorganisms prompted us to elucidate the involvement of glucosinolates in the multifaceted interactions. Glucosinolate containing extracts from 3-day-old *Camelina* seedlings were tested for their effects on the growth of *P. aurantiogriseum*. As determined by UHPLC-MS, the sterilized aqueous *Camelina* extract, prepared by redissolving of the dried methanolic extract in water, contained large amounts of the glucosinolates glucoarabin, glucocamelinin and 11-(methylsulfinyl) undecyl-glucosinolates (Figure 3 and Figure S3). The glucosinolates are by far the dominant secondary metabolites in these *Camelina* extracts, but we could not identify glucosinolate degradation products. The extracts also contain phenolic compounds as minor constituents. The identified phenolic compounds are listed in Table S1.

When exposed to the above described *Camelina* extract in PVK medium, *P. aurantiogriseum* did not degrade the glucosinolates and grew like the controls, while *P. olsonii* grew better in presence of the extract (Figure S2). When *P. aurantiogriseum* was treated with myrosinase-supplemented *Camelina* extracts to release isothiocyanates, the fungus did not grow at all (Figure 3). Therefore, *P. aurantiogriseum* is not inhibited by intact glucosinolates, but it is sensitive to isothiocyanates or other breakdown products.

The finding that *P. aurantiogriseum* did not degrade glucosinolates from *Camelina* or other plants, except for sinigrin (Figure S4), prompted us to examine soil- and root-colonizing bacteria for their ability to degrade the glucosinolates. The four soil bacteria belonging to the genera *Paenibacillus spec*. (BS1-DS), *Arthrobacter spec*. (BS5-DS), *Rhodococcus spec.* (BS2-DS), *Pseudomonas spec*. (BS4-DS), all efficient in phosphate solubilization (Figure 3), were exposed to glucosinolate containing *Camelina* extract for 3 days in PKV medium. UHPLC-MS analyses of the culture media disclosed different capacities of glucosinolate degradation. The *Pseudomonas*, *Arthrobacter* and the *Rhodococcus* species showed highest degradation capacity, while the *Paenibacillus* species degraded less than 60% of the glucosinolates (Figure 3). When *Camelina* seedlings were directly extracted with water instead of methanol, and the sterilized extract was immediately applied to *P. aurantiogriseum* cultures, fungal growth was also inhibited (Figure S2), due to the active myrosinase in this extract. Although the P-solubilization capacity of the inoculants *T. viride* and *P. laurentiana* were unexpectedly low, the growth behavior in the presence of *Camelina* glucosinolates and myrosinase was studied. The *T. viride* strain did not degrade the glucosinolates, but growth was inhibited when myrosinase was added (Figure S5). These findings correspond to the behavior of *P. aurantiogriseum*. The growth of *P. laurentiana* was inhibited during the first 24 hours to about 50% by *Camelina* glucosinolates in the presence of myrosinase (Figure S5). Thus, both strains used for inoculation did not meet our expectation of being glucosinolate tolerant.

The microorganisms collected from the *Camelina* root surface by bathing were also examined for glucosinolate degradation under phosphate deficiency, but these microorganisms did not degrade gluosinolates, and the addition of *P. aurantiogriseum* mycelium did not elicit the degradation. Thus, the root-colonizing microorganisms are not affected by glucosinolates released by the seedlings.

### 2.7. Cyclo (Leu-Pro) Impairs a Camelina Root Colonizing Bacterial Consortium and Represses P. olsonii

The deadly impact of *P. aurantiogriseum* on most of the older *Camelina* seedlings under the phosphate-deficient condition challenged us to identify fungal allelochemicals that may interact with the low amounts of residual glucosinolates released by these plants. Further hints for phytotoxic allelochemicals released by *P. aurantiogriseum* were disclosed by incubations of *Camelina* seedlings with fractions of the *P. aurantiogriseum* culture medium and by coculturing of *Camelina* seedlings with the fungus on Phytoagar (Figure S2).

**Figure 4:**
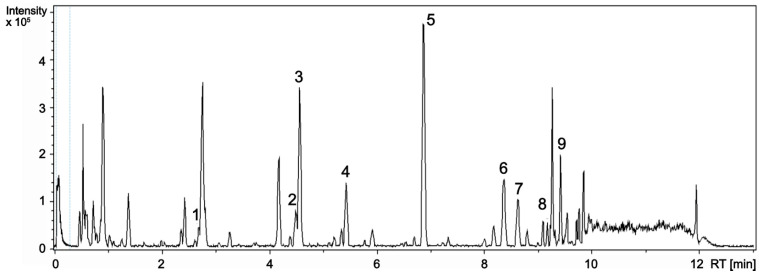
Bioactive metabolites produced by *P. aurantiogriseum*. UHPLC chromatogram of a concentrated ethyl acetate extract of culture medium (*P. aurantiogriseum*, cultured for four weeks). 1: maculosin (cy-clo(L-Pro-L-Tyr); 2: penicillic acid; 3: cyclo(Leu-Pro); 4: cyclo(Phe-Pro); 5: aurantiamine; 6: patulin; 7: auranthin; 8: verrocosidin; 9. fuegin.

*P. aurantiogriseum* produces a variety of bioactive metabolites, which are released into the surrounding environment. Their characterization was achieved by extraction of the *P. aurantiogriseum* culture medium with ethyl acetate followed by UHPLC-HR-MS analysis (Figure 4, Table S2). Data processing with Metabos-cape^®^ allowed the identification of nine compounds of which five have been described to be produced by *P. aurantiogriseum* [27,28] 12: Patuline and penicillic acid, both known for their phytotoxicity, as well as the mycotoxines verrrucosidin, aurantiamine, and auranthine. Furthermore, the bicyclofarnesol derivative fuegin, a sesquiterpene was identified. In addition, we found the antibiotic diketopiperazines (cyclo-dipeptides) cyclo(L-Phe-L-Pro), cyclo(L-Leu-L-Pro), which was most abundant, and maculosin (cyclo(L-Pro-L-Tyr). As diketopiperzines were not yet identified in *P. aurantiogriseum*, cyclo (Leu-Pro) was tested for effects on the yellow- colored bacterial consortium that colonizes *Camelina* roots and which was highly efficient in phosphate solubilization.

**Figure 5:**
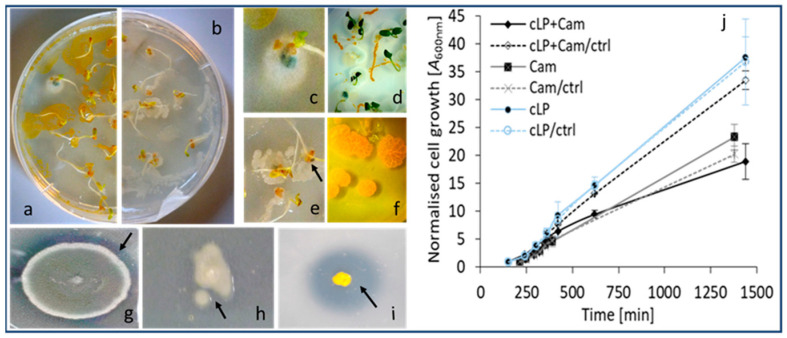
Phosphate solubilization and effect of cyclo(L-Leu-L-Pro) on the microbial consortium of *Camelina* seedlings. ((**a**,**d**): different controls), *Camelina* seedlings on PVK agar with developing colonies of the yellow bacterial consortium (enlargement of colonies shown in (**f**). (**b**) *Camelina* seedlings on PVK agar in the presence of cyclo(L-Leu-L-Pro); no colonies of the yellow consortium are visible, instead, only white *P. polymyxa* colonies are produced (colonies enlarged in (**e**), arrow: empty seed coats). Seed coat-colonizing microorganisms: *P. olsonii* (**c**) grows together with the yellow colonies. *P. olsonii* (**g**), *P. polymyxa* (**h**) and the yellow bacterial consortium (**i**), and can solubilize phosphate from apatite with different capacities, as indicated by the halo zone (arrows). Graph (**j**): Suppressive effect of cyclo(L-Leu-L-Pro) (cLP) in combination with a *Camelina* extract on the growth of the yellow microbial consortium. The yellow bacterial consortium was grown in LB liquid medium in the presence of cLP, *Camelina* extract (Cam) or both. Dashed lines represent the three controls (ctrl) without compound addition (s) for cLP + Cam, Cam and cLP. Data are means ± standard deviations of four biological replicates.

When germinating *Camelina* seeds were placed on PVK agar plates and immediately treated with cyclo(L-Leu-L-Pro), the yellow-colored bacterial colonies, which were dominant on control plates without cyclo(L-Leu-L-Pro), were eliminated (Figure 5). Also, the plant growth promoting and phosphate-solubilizing fungus *P. olsonii* was repressed. In contrast, *P. polymyxa* with a lower capacity of phosphate solubilization was not affected. Growth experiments in LB medium revealed that the yellow bacterial consortium was not inhibited by cyclo(L-Leu-L-Pro) and *Camelina* extracts when applied separately and incubated for 1 day. However, a combination of cyclo(L-Leu-L-Pro) and low concentrated (0.1% *w*/*v*) *Camelina* extract inhibited the growth significantly (*p* < 0.001), down to about 50% (Figure 5).

These experiments point to interactions of *Camelina* together with its associated microorganisms on the one side, and the soil fungus *P. aurantiogriseum* on the other side, are accompanied by the release of allelopathic secondary metabolites by each partner for the suppression of competitors. Thus, a released allelochemical can gain new inhibitory potential, when combined with other compounds. Suppression of the yellow bacterial consortium should considerably reduce phosphate solubilization within the rhizosphere, as the consortium forms biofilms at the surface of *Camelina* roots (Figure 5 and Figure S2). The results suggest that *Camelina* glucosinolates and cyclo(L-Leu-L-Pro) from *P. aurantiogriseum* have the potential to reduce the phosphate availability in the rhizophere, thereby changing the microbial biodiversity on *Camelina* roots and damaging the plant.

### 2.8. Green Manure Experiment-Preservation of Fungi and Dynamic Shifts in Microbial Biodiversity due to Loss of Camelina Glucosinolates

To emphasize the significance of *Camelina* glucosinolates in inhibiting fungal growth, homogenized *Camelina* shoots from plants in the anthesis stage were incorporated into an organic farming soil, and the effect on the soil microorganisms was assessed. We anticipated a reduction in negative impacts on fungi when glucosinolate-free shoot material was incorporated into the soil. Due to the low microbial content of the Dikopshof soil, the incorporation was carried out with an organic farming soil abundant in organic matter (Wiesengut soil), which had not been utilized for *Brassica* cultivation for four years [9].

**Figure 6:**
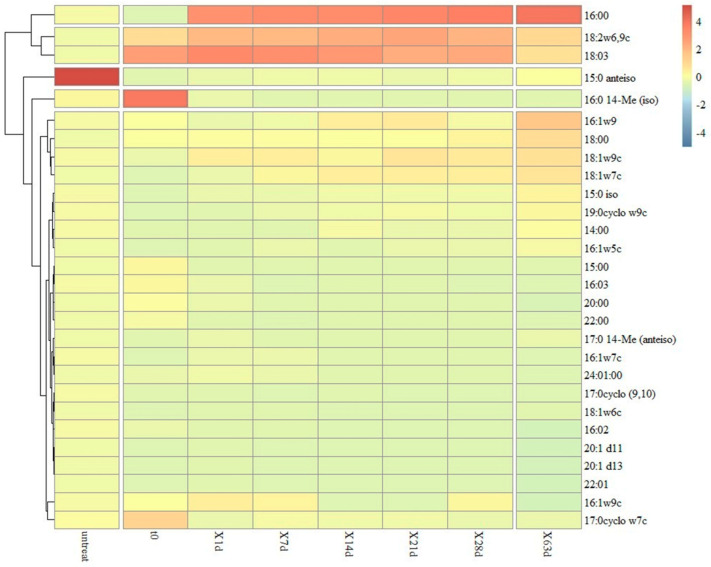
Heat map with cluster dendrogram for visualization of PLFA alterations in soil enriched with *Camelina* shoot material. The fatty acid profile in the untreated soil indicates a high biodiversity of bacterial species in low abundance. Samples were drawn directly after application (t0), then after 1 (X1d), 7 (X7d), 14 (X14d), 21 (X21d), 28 (X28d) and 63 (X63d) days; untreat: untreated soil.

Lipid fingerprinting was employed to estimate the dynamic microbial biodiversity changes. As illustrated in Figure 6, phospholipid-derived fatty acid (PLFA) profiles underwent significant changes upon application of the plant material. A dramatic increase in eukaryotic and microbial fatty acid markers was observed in comparison to the untreated soil shortly after adding the shoot material, which was further intensified during the next day (t1) This indicates (1) the incorporated shoot material contained active associated fungi and bacteria, and (2) the nutrient input may have triggered the activation and proliferation of previously inactive soil microorganisms. In the untreated soil, 15:0 anteiso, a marker for Gram (+) bacteria, was abundant [9]. However, a considerably different profile of PLFAs was established after the application of shoot material at d0, with the highest increase in branched fatty acid 16:0 14-Me. The stress marker 17:0 cyclo of Gram (−) bacteria increased [9], 18:3 showed a strong increase, and the fungal marker 18:2ω 6,9c also increased. The 15:0 anteiso marker disappeared and did not recover at any point during the experiment. Over the following weeks, the PLFA profile experienced less dramatic changes, characterized by a continuous increase of 16:0 (d0-d63) and a continuous decrease of 18:3 from d1 to d63. An increase of the fungal marker 18:2ω6,9c occurred from d1 to d28, followed by a decrease to d63. Even after 63 days, the original fatty acid composition of the untreated soil characterized by Gram (+) bacterial markers, was not reestablished. The arbuscular mycorrhizal fungi PLFA marker 16:1ω5c was low and was not significantly affected by the shoot material. The increase in the fungal marker revealed a positive effect of the almost glucosinolate-free *Camelina* shoot material on the abundance of fungi.

According to the clustering shown in the heat map (Figure 6), a total of five behavioral patterns could be identified in soils enriched with *Camelina* shoot material regarding the changes in PLFA profiles. The first cluster includes only the marker 16:0, which shows a consistent increase in soil with time after soil enrichment with *Camelina* shoot material. The second cluster includes the marker 18:3 as well as the fungal marker 18:2ω 6.9c, which increase rapidly on the day *Camelina* shoot material is applied and maintain this trend for a further three weeks before decreasing. The third cluster includes the Gram (+) bacterial marker 15:0 anteiso, abundant only in untreated soil, decreasing as soon as the soil is enriched with *Camelina* shoot material. The fourth cluster includes the branched fatty acid 16:0 14-Me, which is high only when *Camelina* shoot material is applied but diminishes the next day. The remaining PLFA markers all belong to the last cluster and show no clear response to the application of *Camelina* shoot material.

**Figure 7:**
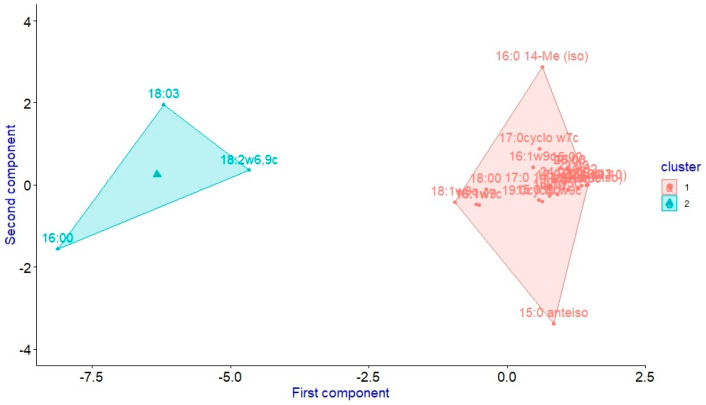
The cluster plot analysis identified two groups of fatty acids. Cluster 1 contains all prokaryotic fatty acids, cluster 2 eucaryotic fatty acids.

Pearson correlations among examined variables showed that, except for the t0 and untreated soil PLFA fatty acid profiles, the remaining variables are highly correlated to each other, showing Pearson correlation values higher than 0.67 to 0.99 (Figure S6). Apart from the above clustering, we can identify two main groups of fatty acids as shown in Figure 7.

Group 1 encompasses prokaryotic fatty acid 15:0 anteiso (Gram (+) bacteria, untreated soil) and 16:0 14Me (t0) in outer positions—and the mycorrhizal fungal marker. Group 2 is composed of the two eukaryotic fatty acid marker 18:2ω6,9 (fungal marker, plant endophytes) and 18:3 (plants) and the fatty acid 16:0 present in eukaryotic and prokaryotic organisms (compare cluster plots shown in Figure 7 and cluster dendro-gram shown in the heat map (Figure 6). Principal component analysis is shown in the Supplementary Materials (Figures S7–S10 and Table S3).

## 3. Discussion

The results provide insights on how *Camelina* root-colonizing microorganisms compete with soil-inhabiting ones for phosphate resources by which allelopathic secondary metabolites are of crucial importance. Glucosinolates and their breakdown products are key allelochemicals involved in the interactions as they preferentially inhibit the growth of soil fungi. The green manure experiment indicates the loss of fungicidal effects in the Wiesengut soil when plant material almost free of glucosinolates was incorporated. Subsequently, an enhanced species diversity of microorganisms including fungi was stated, most of them thought to be originated from the shoot material. This result is considerably different to impacts occurring after applying glucosinolate breakdown products to the Wiesengut soil which has deleterious impacts on fungi, formerly demonstrated by Siebers et al. [9].

Since the Dikopshof soil had no P fertilization for almost 100 years [29], *P. aurantiogriseum* and bacteria of the *Pseudomonas, Rhodococcus, Paenibacillus*, and *Arthrobacter* genera are adapted to low P conditions. All of them solubilize phosphate from apatite. According to the pot experiment with Dikopshof soil, the microorganisms contribute to the phosphate supply of the young *Camelina* seedlings, explaining why their elimination by soil sterilization led to growth stagnation of *Camelina*. However, the high mortality of the seedlings soon after planting in the rhizotron and pot experiments also points to toxic interactions. Bacterial conversions of *Camelina* glucosinolates to less toxic degradation products would weaken the plant´s defense against sensitive competitors and favor, for instance, the growth of *P. aurantiogriseum*. In contrast, soil bacteria contribute to the suppression of fungi, when they degrade the glucosinolates to fungicidal products. On the other hand, *Camelina* glucosinolates have an unexpected harmful effect by acting in concert with cyclo (L-Leu-L-Pro), one of the compounds released by *P. aurantiogriseum*. The combination suppresses the yellow bacterial consortium, the most powerful phosphate solubilizer we could identify in this study. Thus, combinations of secondary metabolites can have other effects than the single compounds. In addition, the arsenal of *P. aurantiogriseum* contains many bioactive secondary metabolites directly toxic to plants, for instance penicillic acid or patulin. It must be expected that all the interfering organisms release secondary metabolites for defense, manipulation, and elimination of competitors, or contrarily, for the promotion of partners, whether as symbionts or as members of microbial assemblies. As mentioned, the quality and quantity of the compounds is influenceable by P availability. Most of the relevant research dealing with molecular effects on a plant consider one microorganism without considering interactions with others. Only more recently, interferences, such as antagonisms, are focused, while secondary metabolites are seldom included [30]. Therefore, we would like to give some examples for several, mainly nonvolatile, secondary metabolites hitherto found to be synthesized by the here addressed microbial taxa in order to open the view for the manifold possibilities of chemical interactions that can occur between the organisms.

*Dikopshof soil bacteria:* Diketopiperazine cyclo(L-Leu-L-Pro), released by *P. aurantiogriseum*, is also produced by several bacteria, for instance *Streptomyces fungicidicus* [31], and *Pseudomonas putida* [32]. Interestingly, the compound suppresses biofilm formation of some Gram (+) bacteria and has nematocidal activities [33,34]. Amino acid based bioactive secondary metabolites are synthesized by many microorganisms. *Arthrobacter spec*. produces the cyclic depsipeptide arthroamide, which inhibits quorum sensing [35]. Species of the genus *Rhodococcus* synthesize multiple bioactive secondary metabolites belonging to different chemical classes, among them nonribosomal peptides, polyketides, and the auxin-related molecules indole-3-acetaldehyde and indole-3-acetic acid. *Rhodococcus species* have also a great capacity to detoxify and to degrade natural and synthetic xenobiotics [36,37]. Precursor compounds for biosynthesis are sometimes from other organisms or compounds released by one species can be converted by another one. As found with the two Dikospshof soil habitants *Pseudomonas spec*. and *Arthrobacter spec.*, also *Rhodococcus spec.* efficiently degrades *Camelina* glucosinolates, while *Paenibacillus spec*. was less successful, almost comparable to *P. aurantiogriseum*.

*The inoculants*: For *Trichoderma species*, more than 200 bioactive secondary metabolites are described [38], for instance nonribosomal peptides, diketopiperazines-like gliotoxin and gliovirin, polyketides, terpenes, and others. Other microorganisms, sometimes prey fungi, influence the production of secondary metabolites in *Trichoderma* species [37]. Gliotoxin synthesis by *Trichoderma viride* is known since 1944 [38].

The failed root colonization of the *T. viride* strain could be caused by glucosinolate degradation products generated by soil bacteria. ITCs, the best studied degradation products, are known to inhibit the root colonization of several *Trichoderma* strains. The isothiocyanate sensitivity of *T. viride* strains was first reported in 1967 [39,40]. Strain-specific positive or negative effects on plants are known from many *Trichoderma* species [41]. Presently, it is unclear, which degradation products (ITCs, nitriles, desulfoglucosinolates or their downstream degradation products) are generated by the Dikopshof subsoil bacteria. Some nitriles have fungicidal activity lower than ITCs but the effects of these compounds are much less investigated. Meanwhile, many further degradation products deriving from isothiocyanates, thiocyanates and nitriles have been identified with unknown bioactivities [42].

The *T. viride* strain used in this study has plant protecting properties but is sensitive to the glucosinolate degradation products, which explains in part the results of the pot experiments with differently aged *Camelina* seedlings. The positive effects of *T. viride* on older *Camelina* seedlings could be due to the release of antifungal compounds for suppressing fungal pathogens such as *P. aurantiogriseum*, of phytohormones and other plant growth stimulatory compounds [43]. The release of plant growth promoting compounds is also suggested for the *A. elegans* consortium, which is highly robust against toxins and seems not to produce toxins in detectable amounts, at least when grown in liquid culture [26].

The inhibitory effect of *Camelina* ITCs on *P. laurentiana* during short-term incubation was not known, and additional studies are necessary to elucidate long-term effects on the growth behavior. Dominant secondary metabolites of the *Pseudomonas* species are non-ribosomal peptides (see above), bacteriocins, N-acetylglutaminylglutamine amides and ß-lactones. Inoculating young seedlings with *T. viride* and *P. laurentiana* seems to complicate the situation as root microbiomes become imbalanced because they are confronted with two further competitive species, in addition to the soil microorganisms. It is known that inoculations with phosphate-solubilizing bacteria alter or even decrease the biodiversity of rhizophere bacteria [44], which supports our assumption.

*Microorganisms associated with Camelina*: As described, we isolated a yellow bacterial consortium harboring a *Cytobacillus firmus* strain, *Paenibacillus polymyxa* and *P. olsonii* from *Camelina* root or seed coats. The bacteria *C. firmus* and *P. polymyxa* have plant growth promoting properties. *Cytobacillus firmus*, formerly *Bacillus firmus* [45], releases indole-3-acetic acid (IAA), produces cyclic surfactant and linear lipopeptides, and nonribosomal peptides, all with antibiotic properties. Other secondary metabolites are polyketide-derived compounds and dihydroisocoumarins. *P. polymyxa*, well known for its plant growth-promoting properties, belongs to the core microbiome of many crops and can also occur as an endophyte [46]. On the other hand, the species can have also inhibitory effects on plants. Plant hormones (indole-3-acetic acid), the peptide antibiotics polymyxins and fusaricidins, antibiotic nonribosomal peptides, and, according to a genome study, probably polyketides, are important secondary metabolites of *P. polymyxa*. Polymyxins disrupt the plasma membrane integrity and causes leakage and are therefore thought to be useful to control plant pathogens. Polymyxins are inhibitory for *Pseudomonas* species [47,48].

*P. olsonii* is a plant growth promoting fungi that produces IAA and ameliorates phosphate supply by phosphate solubilization. Some strains have an endophytic lifestyle, for instance *P. olsonii* ML37, an endophyte in wheat leaves [49]. The secondary metabolites asperphenamate and N-benzoylphenylalanine seem to be produced only in planta [50]. The polyketide xanthoepocin, is widespread in the genus *Penicillium*.

Many of the known compounds are not specific for one species and, most important, strains of the same species can differ considerably in their secondary metabolite patterns. The possibilities of chemical interactions between microorganisms and with plants are exceedingly large. Moreover, parental microbial and plant secondary metabolites can be modified by exposed organisms, resulting in detoxification or degradation products, or otherwise modified parental compounds, all leading to changed bioactivities.

Only few compounds have been investigated for their functions and molecular targets in plants, for instance: gliotoxin promotes defense reactions in tomato plants [51], cyclo (L-Leu-L-Pro induces disease resistance in *Arabidopsis thaliana* [52] and volatiles and nonribosomal peptides of *P. polymyxa* are reported to be responsible for induced systemic resistance [46].

As stated by Pang et al. [53], the importance of secondary metabolites in molecular communication between interacting organisms is still underestimated, as well as functions of changed secondary metabolite accumulations under nutrient deficiency, which can worsen the negative impacts [54,55]. It is likely that all the microorganisms addressed in this study contribute to the allelochemical interactions. We believe that unbalancing of microbial communities triggers the release of toxins by alarmed species for defense of nutrient resources and habitat. Due to the complexity, dissections of further allelochemical interactions could not be addressed in the present study and disentangling the extraordinarily complex interactions needs further intensive studies. Further studies are directed to the identification of the bacterial strains found in the Dikopshof soil and microbial and plant secondary metabolite exudation when faced with released compounds by other organisms.

## 4. Materials and Methods

### 4.1. Soils for Rhizotron, Pot and Green Manure Experiments

Rhizotrons and pots for pot experiment I were filled with Dikopshof soil [24]. For pot experiment II, a nutrient-deficient artificially mixed and standardized substrate type 0 (0-Erde) obtained from the Werkverband e.V., Germany was used (pH (CaCl_2_) 6.1; water holding capacity: 83.4%; N: <2 mg L^−1^ with <2 mg L^−1^ NH_4_-N and <1 mg·L^−1^ NO_3_-N; P_2_O_5_ 5 mg L^−1^; K_2_O 13 mg L^−1^, 19% TOC). 29–31. For the green manure study, the soil (Wiesengut soil) was used as before [9].

### 4.2. Plant Material

Seeds of *Camelina sativa* (L.) Crantz were harvested from plants grown as described by Hölzl and Dörmann [56]. *Camelina* plants used for soil incorporation were grown in a phytotron (160 μmol m−2 s^−1^ light, 25 °C and 65% humidity). Seeds were placed in soil-filled pots, watered three times a week and fertilized once a week. Shoots were harvested when plants reached BBCH scale 64–73 (late flowering).

For preparing extracts, *Camelina* seedlings were grown hydroponically from seeds on cheesecloth under natural conditions. Three-day-old *Camelina* seedlings were harvested, dried between filter paper, weighed, and extracted with methanol (1:1 *w*/*v*). The homogenate was centrifuged at 20,000 g for 5 min at 4 °C and the supernatant evaporated to dryness. The residue was dissolved in water, the resulting suspension centrifuged at 20,000× *g* for 5min. The supernatant was aliquoted and stored at −20 °C. Before use, thawed portions were sterile filtrated (syringe filter Excalibur, pore size 0.22 µm, Labomedic, Germany). For direct application of myrosinase containing *Camelina* extracts, the extraction was performed with water, the homogenate centrifuged as mentioned, the supernatant sterile filtrated and portions of the filtrate directly added to microbial cultures. The extracts were checked for the presence of intact glucosinolates using the UHPLC/MS method described.

### 4.3. Microorganisms Used for Rhizotron and Pot Experiments

The *T. viride* F-00612 consortium (collection of D.K. Zabolotny, Institute of Microbiology and Virology, National Academy of Sciences, Ukraine) was grown in liquid YEP medium for 48 h and the diluted suspension (OD600 = 0.1) used for inoculations in the rhizotron and pot experiments with Dikopshof soil (DI; 50°48′29′′ N, 6°57′11′′ E). In contrast to culturing on agar, the *T. viride* F-00612 consortium loses associated microorganisms, such as *Enterobacter ludwigii* (accession No MH915584), *E. cloacae* (accession No MH915583), *Acinetobacter calcoaceticus* (accession No MH915582), *Bacillus pumilus* (accession No MH915587), *B. subtilis* (accession No MH915585), and *B. safensis* (accession No MH915586), when grown in liquid medium [57]. When used as a consortium, culturing was performed on Czapek agar.

Seed coat-colonizing *Actinomucor elegans* (accession KM404167, registered at the DMSZ (Deutsche Sammlung von Mikroorganismen und Zellkulturen GmbH, Braunschweig) as strain AbRoF1 used for morphological characterization) established stable associations with *Abutilon theophrasti* root-colonizing microorganisms after reinoculation [58]. The resulting consortium contains among others *Pantoea ananatis* (DSM ID 14-714C) and the yeast *Papilliotrema baii* (DSM 100638) as two of the most viable microorganisms on culture plates.

The *Pseudomonas* species was identified by the DSMZ. The strain (PpSalb ID 20-163 (=HM488364) matches to *Pseudomonas laurentiana* GSL-010 (MG719526.1), 100% identity), DSMZ ID20-163. For the rhizotron experiments, the bacterium was cultured in LB medium. A suspension of OD600 = 0.1 was used for inoculation. Growth curves in LB medium supplemented with *Camelina* glucosinolates and myrosinase started with suspensions of OD600nm 0.001 (Figure S5).

### 4.4. Synthesis of ^33^P Labeled Apatite, Rhizotron Growth Studies and ^33^P–Imaging

^33^P-apatite synthesis was performed according to Wolff et al. [59]. The rhizotron experiments followed roughly the method described by Bauke et al. [24]. ^33^P-apatite was mixed with P-depleted, decalcified Dikopshof soil [24,25], placed into rhizotrons and one 4-day-old Camelina seedling per rhizotron was planted. Roots of the seedlings were inoculated with 20 µL *P. laurentiana* and *T. viride* suspensions, then placed into 12 rhizotrons. Non-inoculated plants were used as controls. Rhizotrons were then placed at an angle of 45° in a climate chamber with a day/night-length of 12 h under a slow transition and a light intensity of 320 μmol m−2 s^−1^ PAR. Temperatures were set to 22 °C and 18 °C, respectively, at a relative air humidity of 50%.

Periodic imaging of rhizotrons with suitable imaging plates (200 × 400; DÜRR NDT GmbH & Co. KG, Bietigheim-Bissingen, Germany) using the Bioimager CR35 Bio (Raytest, Straubenhardt, Germany) started one week later. In the scanning process, the photo-stimulated luminescence intensities were measured, receiving a digital autoradiographic image processed by the standard imager software AIDA Image Analyzer 2D (ELYSIA Raytest, Straubenhardt, Germany).

After 3 weeks, the experiment was finished by harvesting the plants. Plants were separated from soil, dried, and digested (6 h, 180 °C) with 4 mL 65% HNO_3_ in a Loftfield apparatus (Loftfields Analytische Lösungen, Neu Eichenberg, Germany). After dilution and filtration, aliquots were mixed with 10 mL scintillation cocktail (ULTIMA Gold XR, PerkinElmer, Solingen, Germany) and the incorporated radioactivity subsequently measured by a Tri-Carb^®^ 3110TR Liquid scintillation counter (PerkinElmer, Solingen, Germany).

### 4.5. Reisolation of Inoculants from Roots after Rhizotron Experiments

*Camelina* roots from suspension-inoculated plants were removed from the rhizotron soil after decay of radioactivity and soil particles were carefully picked off prior to placing the roots on Czapek, LB or Sabouraud agar plates. After culturing for 14 days at 25 °C in the dark, the plates were examined for *T. viride* and *P. laurentiana* and other colonies.

### 4.6. Identification of the Dominant Fungus and Accompanying Bacteria Present in the Dikopshof Soil

Microorganisms present in the Dikopshof soil were isolated by serial dilution as described in Siebers et al. [9], and cultivated on different media (Sabouraud agar, Pikovskaya agar, Czapek agar). Only a few microorganisms were detected after 2 weeks. Discrete colonies of a dominant fungus were further cultured on Sabouraud agar. A representative plate was used for identification of an assumed *Penicillium* species by the DSMZ. For identification, the ITS rDNA sequences and morphological markers were used. The species was identified as *Penicillium aurantiogriseum* Dierckx (isolate 20-165) with a sequence identity of 100% to the reference (GeneBank accession No AF033476).

Single bacterial colonies were picked as template for touchdown-PCR with the bacterial 16S primers (27f AGAGTTTGATCMTGGCTCAG, 1492r(s) CGGTTGTTACGACTT) as described in Schütz et al. [60]. The PCR products were purified with the NucleoSpin Gel and PCR Clean-up Kit (Macherey& Nagel, Düren, Germany) and sequenced. The bacterial sequences were searched against the NCBI Nucleotide database using BLASTN, leading to the identification of species belonging to the genera *Paenibacillus, Rhodococcus, Arthobacter,* and *Pseudomonas*, (accession numbers: BS1_*Paenibacillus*_sp ON620168; BS2_*Rhodococcus*_sp ON620169; BS4_*Pseudomonas*_sp ON620170; BS5_*Arthrobacter*_sp ON620171). The PCR methods with the corresponding primers were also used for the identification of most viable bacteria harvested from the roots after the rhizotron experiment and culturing on Sabouraud agar. The Gene-Bank accession number of *Pseudomonas* sp. P16.1 is: OR123075, and of *Acinetobacter sp*. P17.2: OR123076 (NCBI Nucleotide database).

### 4.7. Setups of Pot Experiments

For the first pot experiment nonsterilized and sterilized Dikopshof soil was used. 50 g of the soil was thoroughly mixed with 2 g unlabeled, analogously synthesized apatite, filled in adequate pots, and watered until the soil was well moistened. Three *Camelina* seedlings (3 days old) were planted in each pot. Root tips of the seedlings were inoculated with 20 µL *P. laurentiana* and *T. viride* suspensions. The plants were cultured in a phytotron (160 μmol m^−2^ s^−1^ light, 25 °C and 65% humidity) until three leaves were developed and cotyledons became yellowish (14–18 days). The plants were watered every second day and fertilized with P fertilizer at days 8–10 when the first two leaves were unfolded. Shoot length was monitored during the entire culture at days shown in Figure 3. Each experiment was performed with 15 pots per plant species and was repeated three times.

The second, 21-day-pot experiment was performed with 25 g 0-soil mixed with 1 g apatite, filled in pots and watered as described above. Two or three *Camelina* seeds were placed into each pot. After seven days, 0.5 cm^2^ agar plugs covered with mycelia either of the *P. aurantiogriseum*, the *T. viride*—or the *A. elegans* consortium were harvested and incorporated to the soil in the following manner: 5 pots—control (no fungus), 5 pots *P. aurantiogriseum,* 5 pots *P. aurantiogriseum* + *T. viride* consortium, 5 pots *T. viride* consortium, and 5 pots *A. elegans*, (*n* = 3). The plants were watered every second day and fertilized with P fertilizer after germination and then every week. The second set up was repeated with P-containing fertilizer.

### 4.8. Determination of Released Phosphate by Inductively Coupled Plasma Mass Spectrometry (ICP-MS)

*P. aurantiogriseum*, *T. viride* (grown in liquid culture), the *T. viride* consortium, the *A. elegans* consortium and *P. olsonii* from the *Camelina* seed coat were precultivated on Sabouraud agar. The collected washable microorganisms from the root surfaces and all other bacteria were precultured in LB liquid medium. Agar plugs (100 mg) of the fungi and 500 µL of the microorganisms grown in LB medium (OD600nm 0.1) were transferred to flasks containing 15 mL PVK (Pikovskaya medium) and cultured for 6 days at 21 °C in the dark. All incubations were performed in triplicate using material from diverse cultures of the given species. Subsequently, the cultures were filtered and the filtrates centrifuged at 20,000 rpm for 15 min at 4 °C to pellet microbial material and apatite particles. The supernatants were transferred to new tubes and again centrifuged at 20,000 rpm for 10 min. The supernatants of the triplicates were combined, aliquoted (2 mL) and stored at −20 °C until analysis. Aliquots of the PVK medium were treated in the same way as the control. Sample preparation for ICP-MS measurements was performed as follows: After centrifugation, the supernatant was transferred into a round-bottomed 15 mL PFA vial (Savillex, Eden Prairie, USA) and placed on a heating plate at 80 °C to be completely dried down in customer-designed laminar flow box in a clean-room. The dried material was redissolved in a mixture of 1 mL 68% ultrapure HNO_3_ and 0.5 mL 30% H_2_O_2_. The closed vial was heated up at 120 °C on a heating plate for 1 h to dissolve any organic matter. After digestion, the solution was dried again and then redissolved in 1 mL 0.3 M HNO_3_ for further dilution before the determination of P concentration started.

The P concentrations were analyzed by quadrupole inductively coupled plasma mass spectrometry (ICP-QMS, Agilent 7900, Agilent, Bremen, Germany). The measurements were performed after two hours of warm-up time to reduce the P background towards 10 ppb (10 µg/L).

### 4.9. Identification of Glucosinolates and Derived Compounds by UHPLC-MS

For the identification, supernatants of the centrifuged culture media and *Camelina* seedling extracts were used. Screening and quantification were carried out using an ultra-high-performance liquid chromatography (UHPLC)-electrospray-mass spectrometer (MS) consisting of an ACQUITY UPLC system equipped with a Xevo TQ-S triple quadrupole mass spectrometer (Waters, Eschborn, Germany) using a Nucleodur C18 Gravity-SB column (150 × 3 mm, 3 µm; Macherey-Nagel, Düren, Germany) thermostated to 25 °C. The 59 min gradient elution was performed at a flow rate of 1 mL/min with Millipore water (Millipore GmbH, Schwalbach, Germany) with 0.1% formic acid (pH 3.0) as solvent A and acetonitril with 0.1% formic acid as solvent B (VWR International GmbH, Darmstadt, Germany, LC-MS grade). The mass spectra were recorded with an ESCi source in positive and negative full scan mode.

### 4.10. Identification of Secondary Metabolites Released from Penicillium Aurantiogriseum by UHPLC/MS-MS

*P. aurantiogriseum* was cultured for four weeks in Czapek yeast medium. The medium was filtrated and the filtrate centrifuged at 10,000× *g* for 15 min. The supernatant was extracted with ethyl acetate and the organic and aqueous phases evaporated to dryness. The dried ethyl acetate phase was used for compound identification. The dry residue was reconstituted with CH_3_CN/MeOH (3:1) using vortexing/sonication. After centrifugation, the supernatant was filtered using a Socorex^®^ borosilicate glass syringe and 0.2 μm MilliQ Millipore^®^ LCR filters. An aliquot of the filtrate was 10× diluted with MeOH prior to injection (1 μL). 

Chromatographic separation was performed on an UltiMate 3000 UHPLC system from Thermo Fisher Scientific (Waltham, MA, USA) equipped with an Acquity BEH C18 column (2.1 × 100 mm, 1.7 μm, Waters) at a flow rate of 450 μL/min at 30 °C. The mobile phase consisted of H_2_O + 0.1% formic acid (A) and acetonitrile + 0.1% formic acid (B). A gradient elution was performed from 5 to 30% B in 8 min, increase to 100% B in 1 min and flushing at 100% B during 2 min. The UHPLC system was connected to a high-resolution QExactive orbitrap mass spectrometer (Thermo Fisher Scientific) equipped with a heated electrospray ion source. Masses were calibrated below <2 ppm accuracy using the Thermo Fischer Pierce calibration solution. Data were acquired in (+)- and (–)-ESI mode, with a mass range from m/z 100 to 800 and 35,000 resolution. MS/MS experiments were performed with a normalized collision energy of 30 eV.

### 4.11. Cyclo(L-Leu-L-Pro)-Tolerant and Intolerant Microorganisms from Camalia sativa Seed Coats

The fungus associated with *C. sativa* seeds was isolated and cultured on TSB medium. Colonies of the most abundant bacteria growing on Pikovskaya plates untreated/treated with 200 µl 1 mM cyclo(L-Leu-L-Pro) were picked and used as templates for identification as described above. The cyclo(L-Leu-L-Pro)-tolerant species was identified as *Paenibacillus polymyxa* (accession No. *Paenibacillus*_sp ON620175). The intolerant yellow colonies were composed of distinct species, thus presenting a bacterial consortium. One of the species which was most abundant in the young colonies matches several *Pseudomonas* species with identical sequence similarity (accession number: *Pseudomonas_sp* ON620172), whereby *Pseudomonas aeruginosa* could be excluded by PCR analysis using specific primers. Another species that proliferated from aging yellow colonies was identified by the DSMZ. A partial 16SrRNA sequence indicates the strain as most likely belonging to *Cytobacillus firmus* (syn. *Bacillus firmus*, ID 22-142; identity 99.9%). Due to the impoverishment of species and their change in abundance during culturing, it was not yet possible to comprehensively characterize this complex consortium.

The identification of the fungus associated with *Camelina* seed coats was performed by the DSMZ (isolate ID: 22-51). For identification, the ITS rDNA fragment and the partial β-tubulin gene were used as barcodes for sequencing. The species was identified as *Penicillium olsonii* Bainier & Sartory (strain CBS 232.60 Ex-Typus). The sequences were compared with reference sequences (Genbank, MycoID und INDOOR; *Penicillium olsonii* btub EF652020, KAS6229).

### 4.12. Growth Behavior of the Bacterial Consortium in the Presence of Cyclo(L-Leu-L-Pro) and Camelina Glucosinolate Containing Extract

Bacterial consortium cultures were inoculated in LB medium to an OD600nm 0.1, grown at 37 °C for 150 min, followed by the addition of (a) cyclo(L-Leu-L-Pro), 1.33 mM in methanol; (b) *Camelina* extract, 0.1% (*v*/*v*) in methanol; and (c) cyclo(L-Leu-L-Pro), 1.33 mM in methanol and *Camelina* extract, 0.1% (*v*/*v*) in methanol. Growth was normalized to OD600nm at which compounds were added to the culture.

### 4.13. Microbial Degradation Capacity of Camelina Glucosinolates

*P. aurantiogriseum* was cultured on Sabouraud agar until colonies were 5 cm in diameter, after which 2–3 mg mycelium were removed from the agar and placed into flasks containing 15 mL PVK medium with 300 µL methanolic *Camelina* extract redissolved in water. The addition of *Camelina* extract was repeated after 3 days of culturing for 6 days at 21 °C in the dark. Cultures were grown in triplicates. After 6 days, the cultures were filtrated, the filtrates centrifuged at 20,000 g for 10 min and the supernatants combined, resulting in one sample. Further cultures using the same designs were supplemented with three units myrosinase (from *Sinapis alba*, Merck, Germany) every two days or with 500 µL of *Camelina* root-colonizing microorganisms (RCM), which were prepared as mentioned above. *Camelina* root-colonizing microorganisms without the fungus were evaluated by inoculation of 15 mL PKV medium with 500 µL of the LB precultured assembly for 6 days under the same conditions as described. All samples were aliquoted and stored at −20 °C until analysis which were performed by UHPLC-MS/MS as described above.

### 4.14. Green Manure Experiment with Camelina Shoot Material

Wiesengut soil (180 g) was filled in a 200 mL glass beaker. *Camelina* above-ground plant material (phenological growth stage: anthesis, BBCH scale: 64–73) was crushed with a homogenizer (Philips HR 2870/50 Minimixer) after adding water in a ratio 1:1 (plant/water (*w*/*v*)). Fifty grams of homogenized plant material were added to the soil resulting in a ratio of 0.28g plant material/g soil) and thoroughly mixed. The beakers were closed with a glass lid and Parafilm and stored in the dark at 21 °C. Samples were drawn directly after application (t0), and then after 1, 7, 14, 21, 28, and 63 days. Treatments were carried out in three biological replicates.

### 4.15. PLFA Analysis

The microbial community structure was described by analysis of the phospholipid fatty acid composition (PLFA) in the soil. Lipid were extracted according to Bligh and Dyer [61] and analyzed as described in Kruse et al. [62]. Lipids were fractionated according to their polarity by solid phase extraction [63]. Soil samples were dried for 24 h at 105 °C in the oven to determine the soil moisture content and dry weight. For PLFA analysis, the acyl groups of the separated lipids were cleaved and converted into their methyl esters by methanolysis (FAMEs), [64]. For quantification, an internal standard (100 µL of tridecanoic acid, 50 µg/mL in methanol) was used. All chemicals were of analytical grade. In total 28 PLFAs were identified in the soil samples. Specific PLFAs (or combinations thereof) were assigned to certain groups of organisms as described in Siebers et al. [9]. For plant material and other eukaryotes such as algae, PLFA 18:3 was used as marker. FAMEs were analyzed using an Agilent 7890 gas chromatograph with Supelco SP-2380 capillary column and a flame ionization detector.

### 4.16. Statistics

Statistical analysis of growth and phosphate data was performed with PRISM 9.0. Significant differences were calculated by use of the student’s t-test. Variables were subjected to one-way analysis of variance (ANOVA). Normality was tested according to Anderson-Darling, DÁgostino & Pearson, Shapiro-Wilk and Kolmogorow Smirnov. Student’s t-tests were performed in addition and *p*-values provided in results. Results are presented in the figures as means ± standard deviation.

To evaluate the effects of green manure application on microorganism, we used clustering and a clustered heatmap to reveal hierarchical clusters in data matrices [65]. To this end, we used heatmap and factoextra packages in R. In addition to the clustering and clustered heatmap, we also performed principal component analysis (PCA) and then both the observations and the original variables were illustrated in the principal component space [66]. In a biplot, closely aligned variables are positively correlated with each other where the stronger the correlation is when the larger the arrows are. Negatively correlated variables are aligned in opposite directions and the strength of the correlation is again measured by the magnitude of the arrows. Noncorrelated variables are typically shown by arrows that are aligned in 90 degrees to each other.

## 5. Conclusions

In conclusion, the results indicate that microorganisms already associated with the roots considerably contribute to phosphate solubilization, arising conflicts with soil microorganisms and those used for inoculation under phosphate-deficient conditions. Exemplified with *P. aurantiogriseum* and young *C. sativa* seedlings, secondary metabolites have a pivotal role in damaging or even killing of competing organisms and of those which release fungicidal compounds. Such functions are presently underestimated for most of the secondary metabolites in agriculture. However, they may contribute to the contradictory effects of phosphate-solubilizing microorganisms when used as biofertilizers in agriculture [67]. The presented study provides an idea of the importance of secondary metabolites in establishing alliances and in manipulation of accompanied organisms, which certainly include alterations of their epigenetics, transcriptomics, and proteomics. In further studies, impacts of released secondary metabolites on coexisting microorganisms and plants under different environmental condition should be considered.

## Data Availability

The datasets generated and/or analyzed during the current study are available from the corresponding author upon reasonable request.

## Supplementary Materials

The following supporting information can be downloaded at: https://www.mdpi.com/article/10.3390/plants12152815/s1, Figure S1: Microorganisms isolated from Dikopshof soil. Figure S2: Phosphate solubilization of *Penicillium aurantiogriseum* in the presence of the glucosinolates and additional information. Figure S3: Identification of *Camelina sativa* glucosinolates. Figure S4: Allelopathic plant-microbe interaction and phosphate solubilzation. Figure S5: Effects of *Camelina* glucosinolates/myrosinase on inoculants. Figure S6: Pearson correlation values higher than 0.67 to 0.99Nd addition information. Figure S7: Principal Component analysis and additional information. Figure S8: The eigenvalues. Figure S9: The cos2 values. Figure S10: Illustration of the contribution (in percent) of the examined variables (untreat, t0 …t63) on PC1 and PC2. Table S1: Identification of major phenolic secondary metabolites; Table S2: *Penicillium aurantiogriseum* compound identification. Table S3: Calculated correlations between examined variables and PC1 and PC2; Additional experiment: Test for the presence of viral plant pathogens in the mycelium of *P. aurantiogriseum*.

## References

[1] Gimsing A.L., Kirkegaard J.A. (2009). Glucosinolates and biofumigation: Fate of glucosinolates and their hydrolysis products in soil. Phytochem. Rev..

[2] Tierens K., Thomma B., Bari R., Garmier M., Eggermont K., Brouwer M., Penninckx I., Broekaert W., Cammue B. (2002). ESA1, an *Arabidopsis* mutant with enhanced susceptibility to a range of necrotrophic fungal pathogens, shows a distorted induction of defense responses by reactive oxygen generating compounds. Plant J..

[3] Hu P., Wu L., Hollister E.B., Wang A.S., Somenahally A.C., Hons F.M., Gentry T.J. (2019). Fungal community structural and microbial functional pat-tern changes after soil amendments by oilseed meals of *Jatropha curcas* and *Camelina sativa*: A Microcosm Study. Front. Microbiol..

[4] Hansen J.C., Schillinger W.F., Sullivan T.S., Paulitz T.C. (2020). Decline in soil microbial abundance when camelina introduced into a monoculture wheat system. Front. Microbiol..

[5] Fahey J.W., Zalcmann A.T., Talalay P. (2001). The chemical diversity and distribution of glucosinolates and isothiocyanates among plants. Phytochemistry.

[6] Andini S. (2020). Antimicrobial Isothiocyanates from Brassicaceae Glucosinolates: Analysis, Reactivity, and Quantitative Structure-Activity Relationships. Ph.D. Thesis.

[7] Berhow M., Vaughn S., Moser B., Karakci D., Polat U. (2014). Evaluating the Phytochemical Potential of *Camelina*: An Emerging New Crop of Old World Origin. In: Jetter, R. (eds) Phytochemicals—Biosynthesis, Function and Application. Recent Adv. Phytochem..

[8] Czerniawski P., Piasecka A., Bednarek P. (2021). Evolutionary changes in the glucosinolate biosynthetic capacity in species representing *Capsella, Camelina* and *Neslia* genera. Phytochemistry.

[9] Siebers M., Rohr T., Ventura M., Schütz V., Thies S., Kovacic F., Jaeger K.-E., Berg M., Dörmann P., Schulz M. (2018). Disruption of microbial community composition and identification of plant growth promoting microorganisms after exposure of soil to rapeseed-derived glucosinolates. PLoS ONE.

[10] Hansen J.C., Schillinger W.F., Sullivan T.S., Paulitz T.C. (2019). Soil microbial biomass and fungi reduced with canola introduced into long-term monoculture wheat rotations. Front. Microbiol..

[11] Hiruma K. (2019). Roles of Plant-Derived Secondary Metabolites during Interactions with Pathogenic and Beneficial Microbes under Conditions of Environmental Stress. Microorganisms.

[12] Kopriva S., Gigolashvili T. (2016). Glucosinolate Synthesis in the Context of Plant Metabolism. Adv. Bot. Res..

[13] Jiang Y., Li J., Caldwell C.D. (2016). Glucosinolate Content of *Camelina* Genotypes as Affected by Applied Nitrogen and Sulphur. Crop Sci..

[14] Pant B.D., Pant P., Erban A., Huhman D., Kopka J., Scheible W.R. (2015). Identification of primary and secondary metabolites with phosphorus status-dependent abundance in *Arabidopsis*, and of the transcription factor PHR1 as a major regulator of metabolic changes during phosphorus limitation. Plant Cell Environ..

[15] Frerigmann H., Piotrowski M., Lemke R., Bednare P., Schulze-Lefert P. (2021). A Network of Phosphate Starvation and Immune-Related Signaling and Metabolic Pathways Controls the Interaction between *Arabidopsis thaliana* and the Beneficial Fungus *Colletotrichum tofieldiae*. Mol. Plant-Microbe Interact..

[16] Oliverio A.M., Bissett A., McGuire K., Saltonstall K., Turner B.L., Fierer N. (2020). The role of phosphorus limitation in shaping soil bacterial communities and their metabolic capabilities. mBio.

[17] Yuling Y., Borges G., Masaaki S., Crozier A., Ashihara H. (2012). Effect of phosphate deficiency on the content and biosynthesis of anthocyanins and the expression of related genes in suspension-cultured grape (*Vitis* sp.) cells. Plant Physiol. Biochem..

[18] Nezamivand-Chegini M., Metzger S., Moghadam A., Tahmasebi A., Koprivova A., Eshghi S., Mohammadi-Dehchesmeh M., Kopriva S., Niazi A., Ebrahimie E. (2022). Nitrogen and phosphorus deficiencies alter primary and secondary metabolites of soybean roots. bioRxiv.

[19] Martín J.F. (2004). Phosphate control of the biosynthesis of antibiotics and other secondary metabolites is mediated by the PhoR-PhoP system: An unfinished story. J. Bacteriol..

[20] Van der Molen K.M., Raja H.A., El-Elimat T., Oberlies N.H. (2013). Evaluation of culture media for the production of secondary metabolites in a natural products screening program. AMB Expr..

[21] Jabbran K., Farooq M., Aziz F.T., Siddique K.H.M., Zcheema A., Farooq M., Wahid A. (2013). Allelopathy and Crop Nutrition.

[22] Shen L., Lin W. (2007). Effects of phosphorus levels on allelopathic potential of rice co-cultured with barnyardgrass. Allelopath. J..

[23] Chevrette M.G., Thomas C.S., Hurley A., Handelsman J. (2022). Microbiome composition modulates secondary metabolism in a multispecies bacterial community. Proc. Natl. Acad. Sci. USA.

[24] Bauke S.L., Landl M., Koch M., Hofmann D., Nagel K.A., Siebers N., Schnepf A., Amelung W. (2017). Macropore effects on phosphorus acquisition by wheat roots—a rhizotron study. Plant Soil.

[25] Mertens F.M., Pätzold S., Welp G. (2008). Spatial heterogeneity of soil properties and its mapping with apparent electrical conductivity. J. Plant Nutr. Soil Sci..

[26] Kia S.H., Schulz M., Ayah E., Schouten A., Müllenborn C., Paetz C., Schneider B., Hofmann D., Disko U., Tabaglio V. (2014). *Abutilon theophrastis* defense against the allelochemical benzoxazolin-2(3*H*)-one: Support by *Actinomucor elegans*. J. Chem. Ecol..

[27] Lund F., Frisvad J.C. (1994). Chemotaxonomy of *Penicillium aurantiogriseum* and related species. Mycol. Res..

[28] García-Gómez P., Bahaji A., Gámez-Arcas S., Muñoz F.J., Sánchez-López M., Almagro G., Baroja-Fernández E., Ameztoy K., De Diego N., Ugena L. (2020). Volatiles from the fungal phytopathogen *Penicillium aurantiogriseum* modulate root metabolism and architecture through proteome resetting. Plant Cell Environ..

[29] Wolff J., Hofmann D., Koch M., Bol R., Schnepf A., Amelung W. (2020). Bioavailability and -accessibility of subsoil allocated ^33^P-labelled hydroxyapatite to wheat under different moisture supply. Sci. Rep..

[30] Lopes M.J.S., Dias-Filho M.B., Gurgel E.S.C. (2021). Successful Plant Growth-Promoting Microbes: Inoculation Methods and Abiotic Factors. Front. Sustain. Food Syst..

[31] De Carvalho C.C.C.R., Caramujo M.J. (2018). The Various Roles of Fatty Acids. Molecules.

[32] Li X., Dobretsov S., Xu Y., Xiao X., Hung O.S., Qian P.-Y. (2006). Antifouling diketopiperazines produced by a deep-sea bacterium, *Streptomyces fungicidicus*. Biofouling.

[33] Zhai Y., Shao Z., Cai M., Zheng L., Li G., Yu Z., Zhang J. (2019). Cyclo(l-Pro–l-Leu) of *Pseudomonas putida* MCCC A00316 Isolated from Antarctic Soil: Identification and Characterization of Activity against *Meloidogyne incognita*. Molecules.

[34] Gowrishankar S., Sivaranjani M., Kamaladevi A., Ravi A.V., Balamurugan K., Pandian S.K. (2016). Cyclic dipeptide cyclo(l-leucyl-l-prolyl) from marine *Bacillus amyloliquefaciens* mitigates biofilm formation and virulence in *Listeria monocytogenes*. Pathog. Dis..

[35] Igarashi Y., Yamamoto K., Fukuda T., Shojima A., Nakayama J., Carro L., Trujillo M.E. (2015). Arthroamide, a Cyclic Depsipeptide with Quorum Sensing Inhibitory Activity from *Arthrobacter* sp.. J. Nat. Prod..

[36] Zampolli J., De Giani A., Di Canito A., Sello G., Di Gennaro P. (2022). Identification of a Novel Biosurfactant with Antimicrobial Activity Produced by *Rhodococcus opacus* R7. Microorganisms.

[37] Elsayed Y., Refaat J., Abdelmohsen U.R., Fouad M.A. (2017). The Genus *Rhodococcus* as a source of novel bioactive substances: A review. J. Pharmacogn. Phytochem..

[38] Brain P.W. (1944). Production of Gliotoxin by *Trichoderma viride*. Nature.

[39] Drobnica L., Zemanová M., Nemec P., Kristián P., Antos K., Hulka A. (1967). Antifungal activity of isothiocyanates and related compounds. II. Mononuclear aromatic isothiocyanates. Appl. Microbiol..

[40] Plaszkó T., Szúcs Z., Vasas G., Gonda S. (2021). Effects of Glucosinolate-Derived Isothiocyanates on Fungi: A Comprehensive Review on Direct Effects, Mechanisms, Structure-Activity Relationship Data and Possible Agricultural Applications. J. Fungi.

[41] Pedrero-Méndez A., Insuasti H.C., Neagu T., Illescas M., Rubio M.B., Monte E., Hermosa R. (2021). Why Is the Correct Selection of *Trichoderma* Strains Important? The Case of Wheat Endophytic Strains of *T. harzianum* and *T. simmonsii*. J Fungi.

[42] Plaszkó T., Szúcs Z., Vasas G., Gonda S. (2022). Interactions of fungi with non-isothiocyanate products of the plant glucosinolate pathway: A review on product formation, antifungal activity, mode of action and biotransformation. Phytochemistry.

[43] Tyśkiewicz R., Nowak A., Ozimek E., Jaroszuk-Ścisel J. (2022). *Trichoderma*: The Current Status of Its Application in Agriculture for the Biocontrol of Fungal Phytopathogens and Stimulation of Plant Growth. Int. J. Mol. Sci..

[44] He D., Wan W. (2021). Phosphate-Solubilizing Bacterium *Acinetobacter pittii* gp-1 Affects Rhizosphere Bacterial Community to Alleviate Soil Phosphorus Limitation for Growth of Soybean (*Glycine max*). Front. Microbiol..

[45] Kaspar F., Neubauer P., Gimpel M. (2019). Bioactive Secondary Metabolites from *Bacillus subtilis*: A Comprehensive Review. J. Nat. Prod..

[46] Langendries S., Goormachtig S. (2021). *Paenibacillus polymyxa*, a Jack of all trades. Environ. Microbiol..

[47] Raza W., Yang W., Shen Q.R. (2008). *Paenibacillus polymyxa*: Antibiotics, hydrolytic enzymes and hazard assessment. J. Plant Pathol..

[48] Mohd Din A.R.J., Rosli M.A., Mohamad Azam Z., Othman N.Z., Sarmidi M.R. (2020). *Paenibacillus polymyxa* Role Involved in Phosphate Solubilization and Growth Promotion of *Zea mays* Under Abiotic Stress Condition. Proc. Natl. Acad. Sci. India Sect. B Biol. Sci..

[49] Tarroum M., Romdhane W.B., Al-Qurainy F., Ali A.A.M., Al-Doss A., Fki L., Hassairi A. (2022). A novel PGPF *Penicillium olsonii* isolated from the rhizosphere of *Aeluropus littoralis* promotes plant growth, enhances salt stress tolerance, and reduces chemical fertilizers inputs in hydroponic system. Front. Microbiol..

[50] Rojas E.C., Jensen B., Jørgensen H.J.L., Latz M.A.C., Esteban P., Collinge D.B. (2022). The Fungal Endophyte *Penicillium olsonii* ML37 Reduces *Fusarium* Head Blight by Local Induced Resistance in Wheat Spikes. J. Fungi.

[51] Zaid R., Koren R., Kligun E., Gupta R., Leibman-Markus M., Mukherjee P.K., Kenerley C.M., Bar M., Horwitz B.A. (2022). Gliotoxin, an immunosuppressive fungal metabolite, primes plant immunity: Evidence from *Trichoderma virens*-tomato interaction. MBio.

[52] Noh S.W., Seo R., Park J.K., Manir M.M., Park K., Sang M.K., Moon S.S., Jung H.W. (2017). Cyclic Dipeptides from *Bacillus vallismortis* BS07 Require Key Components of Plant Immunity to Induce Disease Resistance in *Arabidopsis* against *Pseudomonas* Infection. Plant Pathol. J..

[53] Pang Z., Chen J., Wang T., Gao C., Li Z., Guo L., Xu J., Cheng Y. (2021). Linking Plant Secondary metabolites and Plant Microbiomes: A Review. Front. Plant Sci..

[54] Grijseels S., Nielsen J.C., Nielsen J., Larsen T.O., Frisvad J.C., Nielsen K.F., Frandsen R.J.N., Workman M. (2017). Physiological characterization of secondary metabolite producing *Penicillium* cell factories. Fungal Biol. Biotechnol..

[55] Maver M., Trevisan F., Miras-Moreno B., Lucini L., Trevisan M., Cesco S., Mimmo T. (2022). The interplay between nitrogenated allelochemicals, mineral nutrition and metabolic profile in barley roots. Plant Soil.

[56] Hölzl P., Dörmann P. (2021). Alterations of flower fertility, plant size, seed weight, and seed oil con-tent in transgenic *Camelina sativa* plants overexpressing CYP78A. Ind. Crops Prod..

[57] Voloshchuk N., Schütz V., Laschke L., Gryganskyi A.P., Schulz M. (2020). The *Trichoderma viride* F-00612 consortium tolerates 2-amino-3*H*-phenoxazin-3-one and degrades nitrated benzo[*d*]oxazol-2(3*H*)-one. Chemoecology.

[58] Schulz M., Sicker D., Schackow O., Hennig L., Yurkov A., Siebers M., Hofmann D., Disko U., Ganimede C., Mondani L. (2017). Interspecies-cooperations of *Abutilon theophrasti* with root colonizing microorganisms disarm BOA-OH allelochemicals. Plant Signal. Behav..

[59] Wolff J., Hofmann D., Amelung W., Lewandowski H., Kaiser K., Bol R. (2018). Rapid wet chemical synthesis for ^33^P-labelled hydroxyapatite—an approach for environmental research. Appl. Geochem..

[60] Schütz V., Frindte K., Cui J., Zhang P., Hacquard S., Schulze-Lefert P., Knief C., Schulz M., Dörmann P. (2021). Differential Impact of Plant Secondary Metabolites on the Soil Microbiota. Front. Microbiol..

[61] Bligh E.G., Dyer W.J. (1959). A rapid method of total lipid extraction and purification. Can. J. Biochem. Physiol..

[62] Kruse J., Abraham M., Amelung W., Baum C., Bol R., Kühn O., Lewandowski H., Niederberger J., Oelmann Y., Rüger C. (2015). Innovative methods in soil phosphorus research: A review. J. Plant Nutr. Soil Sci..

[63] Gasulla F., vom Dorp K., Dombrink I., Zähringer U., Gisch N., Dörmann P., Bartels D. (2013). The role of lipid metabolism in the acquisition of desiccation-tolerance in *Craterostigma plantagineum*. A comparative approach. Plant J..

[64] Browse J., McCourt P.J., Somerville C.R. (1986). Fatty acid composition of leaf lipids determined after combined digestion and fatty acid methyl ester formation from fresh tissue. Anal. Biochem..

[65] Engle S., Whalen S., Joshi A. (2017). Unboxing cluster heatmaps. BMC Bioinform..

[66] Gabriel K.R. (1971). The biplot graphic display of matrices with application to principal component analysis. Biometrika.

[67] Raymond N.S., Gómez-Muñoz B., van der Bom F.J.T., Nybroe O., Jensen L.S., Müller-Stöver D.S., Oberson A., Richardson A.E. (2020). Phosphate-solubilizing microorganisms for improved crop productivity: A critical assessment. New Phytol..

