# Supplementary material for: Implications of Below-Ground Allelopathic Interactions of Camelina sativa and Microorganisms for Phosphate Availability and Habitat Maintenance
Source: Plants (Basel). 2023 Jul 29;12(15):2815. doi: 10.3390/plants12152815 (PMC10421311; doi:10.3390/plants12152815)
Supplement: Supplementary file 1 [file plants-12-02815-s001.zip › Supplementary information 2 rev.pdf]

**Supplementary Information (SI)**

# Implications of Below-Ground Allelopathic Interactions of *Camelina sativa* and Microorganisms for Phosphate Availability and Habitat Maintenance

Diana Hofmann [1], Björn Thiele [1], Meike Siebers [2], Mehdi Rahmati [3,1], Vadim Schütz [2,†], Seungwoo Jeong [2], Jiaxin Cui [2], Laurent Bigler [4], Federico Held [4], Bei Wu [1], Nikolina Babic [5], Filip Kovacic [5], Joachim Hamacher [6], Georg Hölzl [2], Peter Dörmann [2] and Margot Schulz [2,*]

1. IBG-3: Agrosphäre, Forschungszentrum Jülich GmbH, 52428 Jülich, Germany; d.hofmann@fz-juelich.de (D.H.); b.thiele@fz-juelich.de (B.T.); m.rahmati@fz-juelich.de (M.R.); b.wu@fz-juelich.de (B.W.)
2. IMBIO Institute of Molecular Physiology and Biotechnology of Plants, University of Bonn, 53115 Bonn, Germany; MeikeSiebers@gmx.de (M.S.); v_schuetz@snu.ac.kr (V.S.); s6sejeon@uni-bonn.de (S.J.); s6jicuii@uni-bonn.de (J.C.); hoelzl@uni-bonn.de (G.H.); doermann@uni-bonn.de (P.D.)
3. Department of Soil Science and Engineering, University of Maragheh, 83111 - 55181 Maragheh, Iran
4. Department of Chemistry, University of Zurich, CH-8057 Zurich, Switzerland; laurent.bigler@chem.uzh.ch (L.B.); federico.held@uzh.ch (F.H.)
5. Institute of Molecular Enzyme Technology, Heinrich-Heine-University of Düsseldorf and Forschungszentrum Jülich GmbH, 52428 Jülich, Germany; bnikolina88@gmail.com (N.B.); f.kovacic@fz-juelich.de (F.K.)
6. Plant Diseases and Crop Protection, Institute of Crop Science and Resource Conservation, University of Bonn, 53115 Bonn, Germany; hamacher@uni-bonn.de

* Correspondence: ulp509@uni-bonn.de; Tel.: +49-(0)228 732151
† Present address: Research Center of Plant Plasticity, Seoul National University, Seoul 08826, Korea.

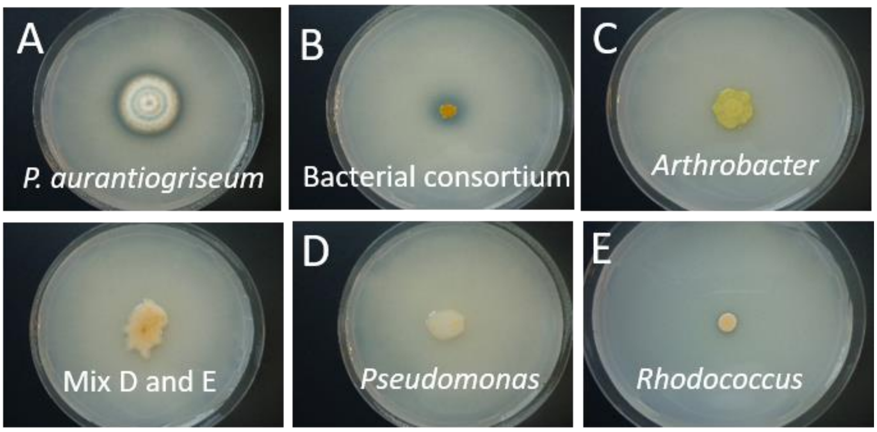


**Figure S1.** Microorganisms isolated from Dikopshof soil. A: *Penicillium aurantiogriseum*; C: *Arthrobacter;* for comparison: B (bacterial consortium) cultured on Pikovskaya (PVK) agar plates. Transparent halo zones indicate a strong phosphate solubilization from apatite. C, D, E and a mixture of D and showed a lower or only weak solubilization compared to the bacterial consortium colonizing *Camelina* roots (see also Figures 5 and 7).

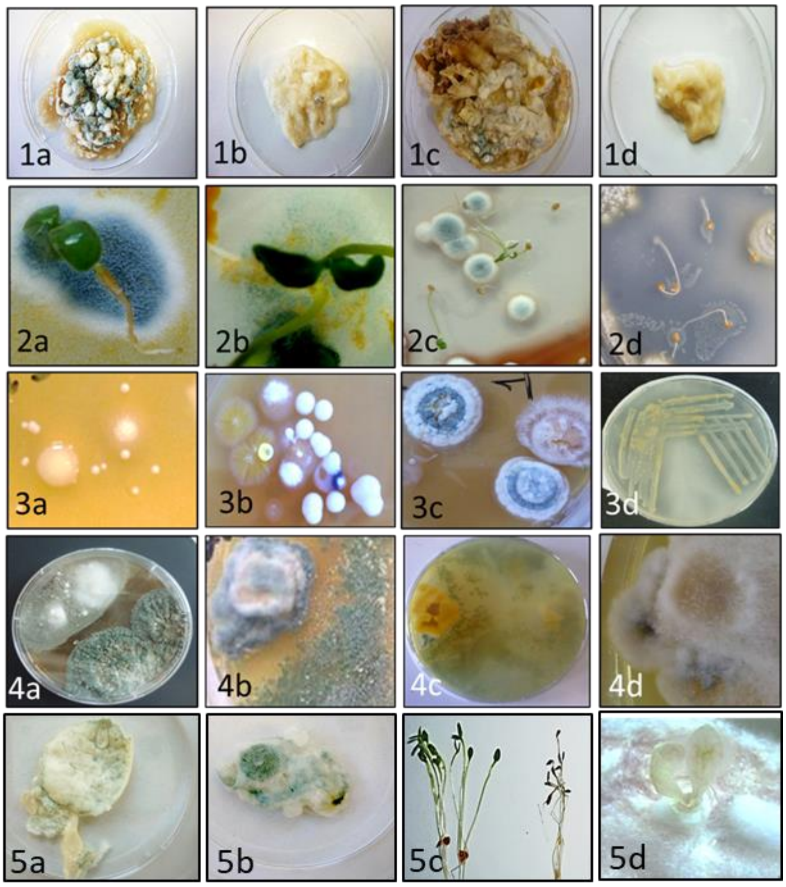


**Figure S2.** Allelopathic Plant-microbe interaction and phosphate solubilzation.

**Panel 1** *Camelina´s* glucosinolate and sinigrin break down products suppress growth of *P. aurantiogriseum*. *P. aurantiogriseum* (start condition: 100 mg mycelium/250 mL medium). (**a**) Liquid PVK medium (3-week-culture: 3WC). (**b**) as (**a**), but with +2 x 10 mg sinigrin, (**c**) liquid Czapek + yeast extract, three-week-culture. (**d**) as (**c**), but with sterilized *Camelina* extract 1 (3 x2 mL). 3WC: three-week culture.

**Panel 2** *Camelina* seedlings on PVK agar. (**a**) 4.day-old seedlings: *P. olsonii* does not damage the seedling and allows growth of a yellow bacterial consortium. (**b**) Co-existence of *P. olsonii*, the bacterial consortium and the *Camelina* seedling after seven days of culture. Culture of *Camelina* seedlings on PVK agar in presence of *P. aurantiogriseum*, (**c**) after three days, the seedling is surrounded by *P. olsonii*, (**d**) after 7 days *Camelina* seedlings died. The mycelium of *P. aurantiogriseum* did not contained plant viral pathogens. Transparent areas on the plates indicate phosphate solubilization from apatite.

**Panel 3**a, b, c, Culturable microorganisms from Dikopshof-subsoil on Sabouraud agar, after 1 week (**a**), after 2 weeks (**b**), after 4 weeks (**c**), *P. aurantiogriseum* has eliminated or overgrown other microorganisms. (**d**) *Camelina* root surface microorganisms on PVK agar. Transparent areas on the plates indicate phosphate solubilization from apatite.

**Panel 4** Co-cultures of fungi. (**a**) stable inhibition zone between *P. olsonii* and *P. aurantiogriseum* (greenish). (**b**) Initial inhibition zone between *T. viride* and *P. aurantiogriseum*, PVK agar. (**c**) Same plate than (**b**), one week later. *T. viride* starts to overgrow *P. aurantiogriseum*. (**d**) *A. elegans* takes seat on *P. aurantiogriseum,* PVK agar.

**Panel 5** *P. olsonii* with *Camelina* extract (5a) and without the extract (5b); 5c: *P. aurantiogriseum*: toxic effect on *Camelina* seedling (right seed, left side control); 5D co-culture of *P. aurantiogriseum* with *Camelina* seedling on phytoagar. *P. aurantiogriseum* kills *Camelina* (decolored).

## II. Secondary Metabolites of *Camelina sativa* and *Penicillium aurantiogriseum*

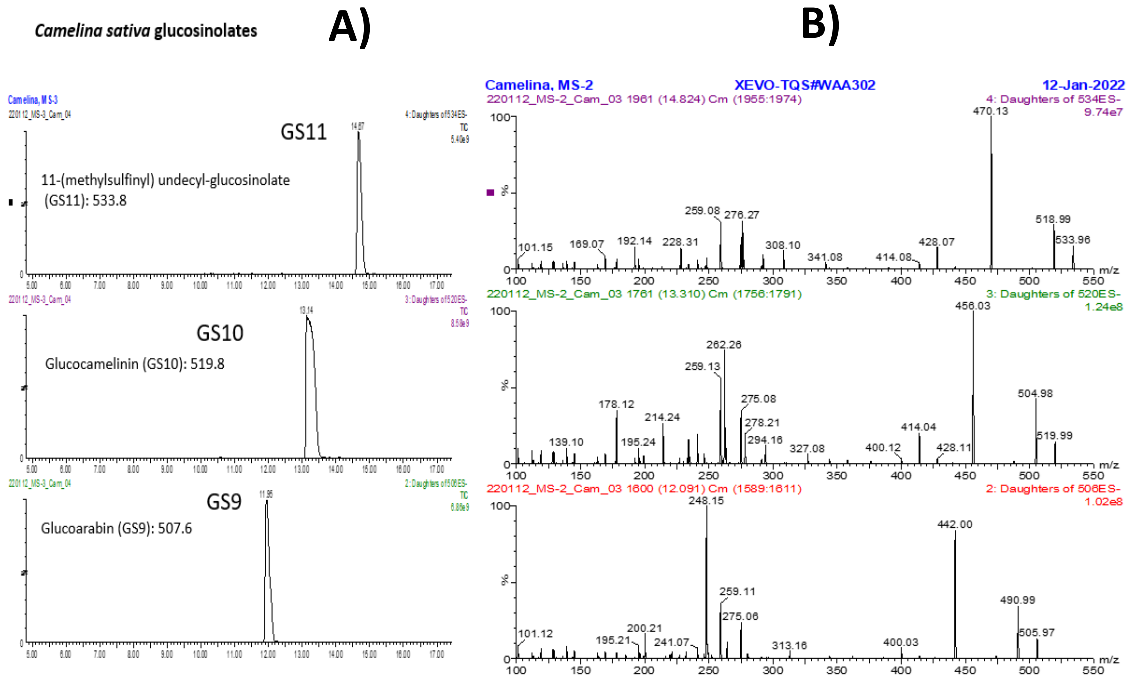


**Figure S3.** Identification of *Camelina sativa* glucosinolates by UHPLC-(–)-ESI-MS. Extracted ion chromatograms of GS11, GS10, and GS9 (A), and corresponding MS/MS spectra (B).

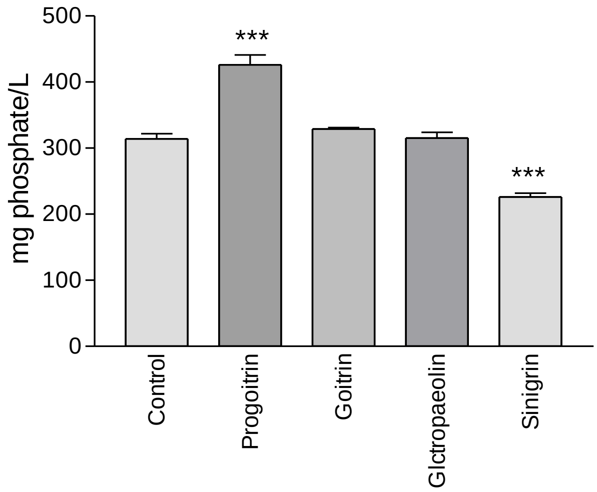


**Figure S4.** Phosphate solubilization of *Penicillium aurantiogriseum* in the presence of the glucosinolates. Progoitrin, glucotropaeolin, sinigrin and the cyclic ITC goitrin in comparison to the control (no added compound). The concentrations were determined by ICP-MS.

### *Cultures of Penicillium aurantiogriseum in presence of glucosinolates and goitrin*

The pre-cultures of fungi were done on Sabouraud Agar. 100 mg agar plugs covered with mycelium were placed in flasks containing 250 ml medium under sterile conditions. After placing, the media were supplemented with either 2x10mg sinigrin within 6 days, or with methanolic *Camelina* extracts dried, redissolved in water and sterilized (3 x 2ml within 6 days). Similarily, the glucosinolates progoitrin, glucotropaeolin and the cyclic ITC goitrin were used for incubations. The cultures were terminated after 14 days, the mycelium harvested by filtration and placed on filter paper to remove liquid prior to

photographic documentation. The media were used for ICP-MS.

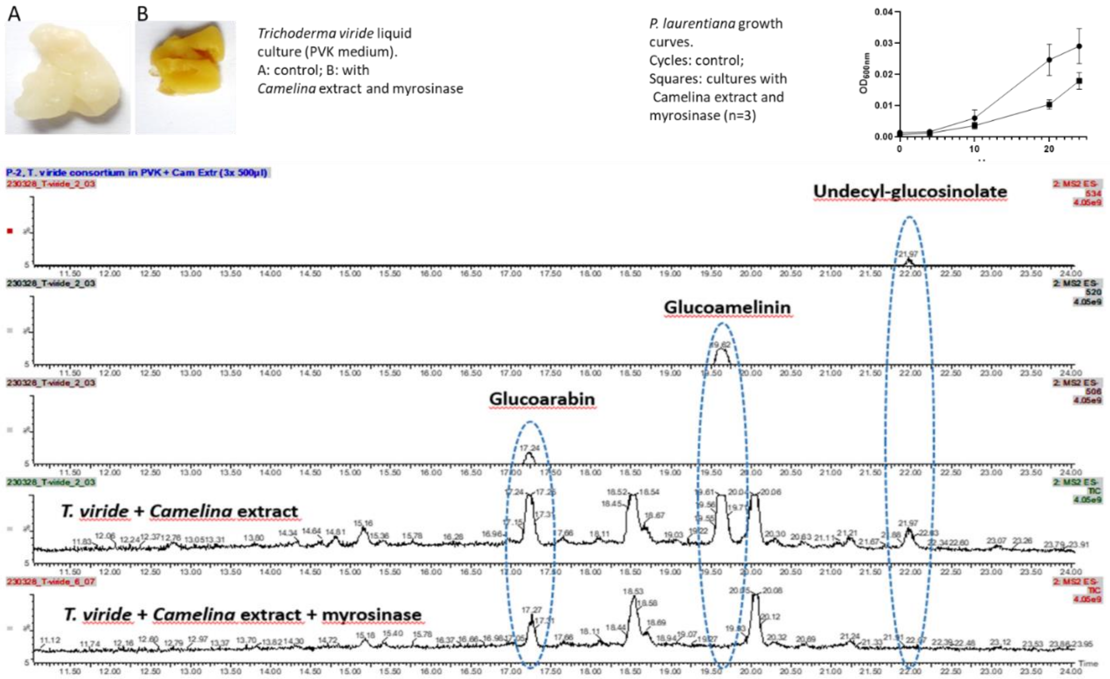


**Figure S5.** Effects of *Camelina* glucosinolates/myrosinase on inoculants.
Pictures A and B: Growth of inoculant *Trichoderma viride* without (control) and in presence of *Camelina* glucosinolates supplemented with myrosinase.
Graph: Inoculant *Pseudomonas laurentiana* growth curve without (control) and in presence of *Camelina* glucosinolates.
The LC-MS chromatograms show the *Camelina* glucosinolates standards, glucocamelinin and undecyl-glucosinolate (marked by dash lines) in the control runs with one of the compounds in *Trichoderma viride* culture medium supplemented with *Camelina* extract and in *Trichoderma viride* culture medium supplemented with *Camelina* extract and myrosinase. When myrosinase was added, undecylglucosinolate, and glucoamelinin are completely degraded, while some glucoarabin is left after 3 days of culture.

**III. Green Manure Experiment**

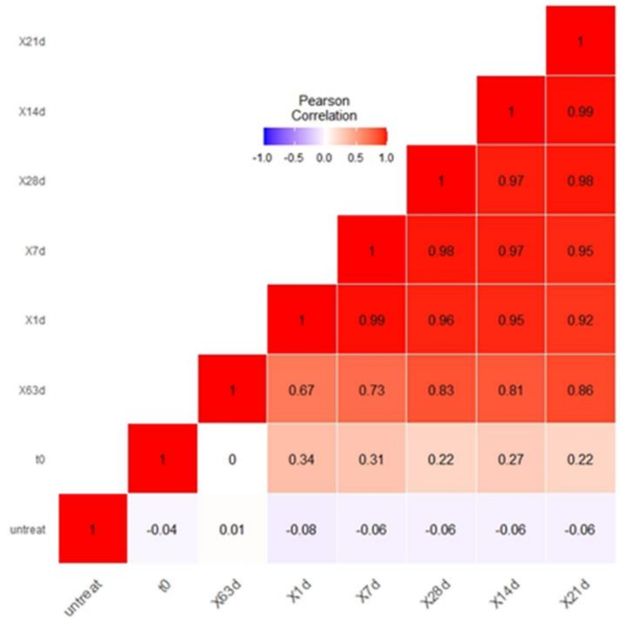


**Figure S6.** Pearson correlation values higher than 0.67 to 0.99.

The PLFA fatty acid data were used for principal component analysis (Fig. S7-S10). The eigenvalues depict that PC1 and PC2 explains more than 83 % of the variation within the data which is efficient to exclude further components from analysis (Fig. S8), square cosine or squared coordinates (cos2) of the fatty acids over PC1 and PC2 are shown in Figure S7. The cos2 values underline the quality of the representation of the fatty acids on the factor map (PC1 and PC2). The cos2 values are higher for cluster 1 than for cluster 2, which indicates that these fatty acids are presented by PC1 and PC2 with enough

quality (Fig S7). The contribution (in percent) of the examined variables (untreat, t0, …t63 = same of the samples) on PC1 and PC2 are illustrated in Fig. S8. All variables show a contribution of 5 to 15 percent on PC1 and PC2 which seems again to be sufficient to keep them in the analysis.

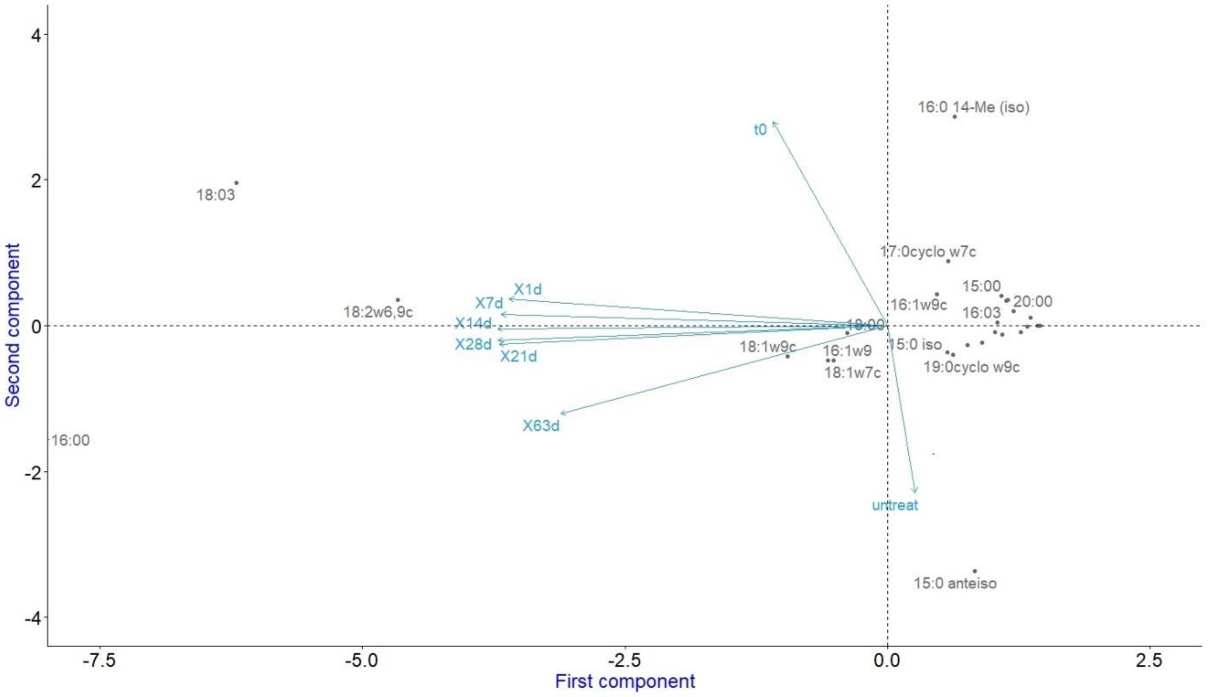


**Figure S7.** Principal Component analysis confirms the differentness of fatty acid compositions determined in untreated, d0 and d63 soil samples by correlations of the variables.

The biplot (Fig.S7) summarizes the relationships between examined variables and PC1 and PC2. As seen from the biplot, x1d, x7d, x14d, x21d, x28d, and x63d are positively correlated with each other (as shown by co-aligned arrows) but negatively with PC1 (as shown with left-aligned arrows). Nearly similar lengths of arrows also indicate that these variables are correlated with PC1 with nearly similar strength. The angles between arrows and PC1 axis also show that these variables are highly correlated with each other except for x63d which is already proved by previously provided heatmap of Pearson correlations between variables. Contrary to PC1, untreat and t0 variables are correlated with PC2. An upward alignment of the arrow in the case of t0 shows that t0 is positively correlated with PC2 while a downward alignment of arrow in the case of untreat variable shows that untreat variable is negatively correlated with PC2. This is also proved by calculating the correlations between examined variables and PC1 and PC2 (Table S3). Following these explanations, PC1 can be used to classify the time-dependent response of the soil to *Camelina* shoot application, as reflected in the changes in PLFA profiles in the days following application. In this way, we can form two groups: 1) one that includes markers 16:00, 18:03, and 18:2w6.9c, which show a strong change after *Camelina* shoot application, and 2) the remaining markers that do not show a clear time-dependent response in the days following *Camelina* shoot application. However, PC2 shows a kind of direct, time-independent, and binary (yes or no) response of the soil to the application of *Camelina* shoot material, which again confirms the results.

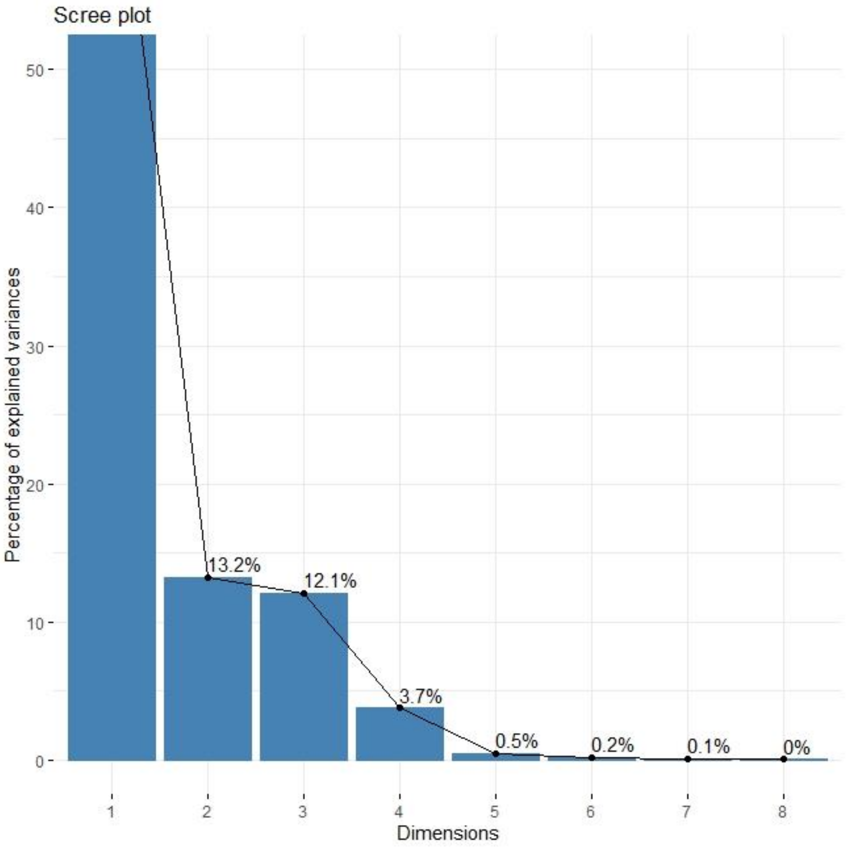


**Figure S8.** The eigenvalues depict that PC1 and PC2 explains more than 83 percent of variation within the data.

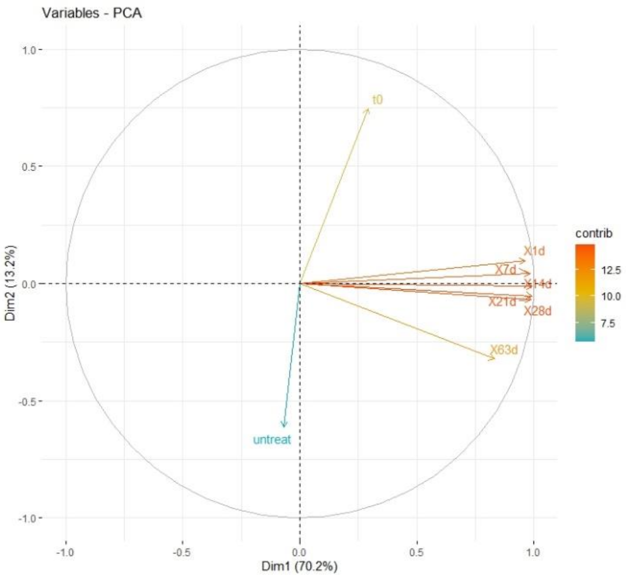


**Figure S9.** The cos2 values underline the quality of the representation of the fatty acids on factor map (PC1 and PC2). The cos2 values are higher for cluster 1 than for cluster 2, which indicates that those fatty acids are presented by PC1 and PC2 with enough quality.

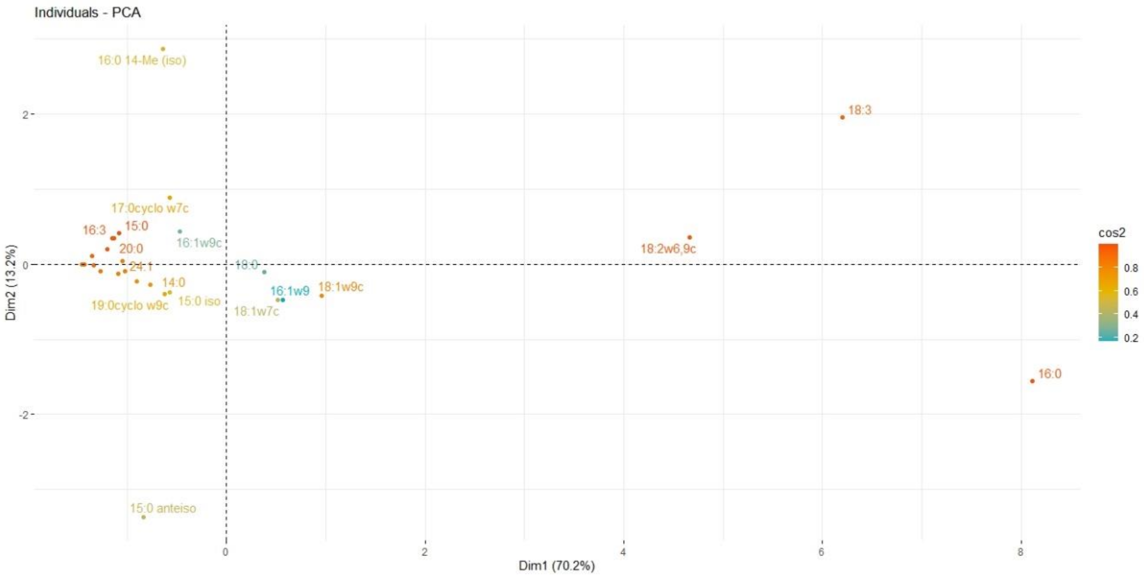


**Figure S10.** Illustration of the contribution (in percent) of the examined variables (untreat, t0 …t63) on PC1 and PC2.

**Table S1:** Identification of major phenolic secondary metabolites and verification of trace amounts of gluconic acids in methanolic extracts from *Camelina* seedlings (gluconic acid is a major compound in *P. aurantiogriseum* culture medium PVK). Some compounds are characterized only in the (–)-ESI mode. The identifications are supported by measurements of reference compounds.

| Compound | Formula | RT [min] | $[M-H]^-$ | Score* | MS/MS fragments | $[M+H]^+$ | Score | MS/MS fragments | Remarks | References |
|---|---|---|---|---|---|---|---|---|---|---|
| Gluconic acid | $C_6H_{12}O_7$ | 2.7 | 195 | 0.96 | 177,129,99,87,85, 75,71,59 | | | | in Cam extracts traces only, one of main components in mixtures with *P. aurantiogriseum* | *https://massbank.eu |
| Quinic acid | $C_7H_{12}O_6$ | 6.9 | 191 | | 93,85 | | | | metabolite/ presursor of chlorogenic acid | |
| Catechinglucosid & Epicatechinglucosid | $C_{21}H_{24}O_{11}$ | 14.3 14.8 | 451 | 0.64 | 289,245,205,203, 179,165,137,125, 109,97 | 453 | 0.46 | 235,205,165,147, 139,123,85 | | https://massbank.eu |
| Chlorogenic acid | $C_{16}H_{18}O_9$ | 15.6 | 353 | 0.88 | 191,161,135,127, 93,85 | 355 | 0.89 | 163,145,135,117, 89 | | https://massbank.eu |
| Sinapoylglucosid | $C_{17}H_{22}O_{10}$ | 16.3 | 385 | 0.88 | 205,190,175,164, 149,89,71 | 387 | 0.54 | 359,342,305,225, 207,175,147,119, 91 | | https://massbank.eu |
| Glucoarabin | $C_{17}H_{33}NO_{10}S_3$ | 18.0 | 506 | | 491,442,400,275, 259,248,241,200, 97 | | | | | Yuan, D et al., (2017) |
| Quercetin-O-Hex-dHex-pen | $C_{32}H_{38}O_{20}$ | 18.2 | 741 | 0.96 | 300 | 743 | 0.41 | 303 | | https://massbank.eu |
| Rutin | $C_{27}H_{30}O_{16}$ | 19.5 | 609 | 0.98 | 300,272,179,151 | 611 | 0.91 | 303 | | https://massbank.eu |
| Glucoamelinin | $C_{18}H_{35}NO_{10}S_3$ | 20.2 | 520 | | 505,456,414,275, 262,259,214,178, 97 | | | | | Yuan, D et al. (2017) |
| Undecyl-glucosinolate | $C_{19}H_{37}NO_{10}S_3$ | 22.7 | 534 | | 519,470,428,308, 276,259,97 | | | | | Yuan, D et al. (2017) |
| Quercetin | $C_{15}H_{10}O_7$ | 27.5 | 301 | 0.85 | 273,179,169,151, 139,121,107,93 | 303 | 0.85 | 285,257,229,201, 153,137,121,109 | | https://massbank.eu |

Structures

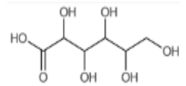
Gluconic acid

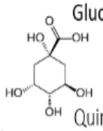
Quinic acid

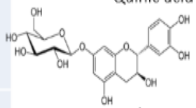
Epi-/catechin

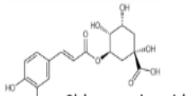
Chlorogenic acid

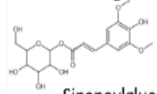
Sinapoylglucoside

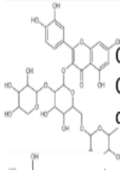
Quercetin-O-Hex-dHex-pen

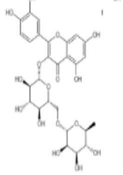
Rutin

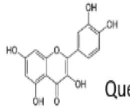
Quercetin

## **Table S2** *Penicillium aurantiogriseum* compound identification by UHPLC-MS/MS

| Compound | Peak Nr. | RT [min] | m/z [M+H]$^+$ | Formula | m/z$_{theo}$ [M+H]+ | Deviation [ppm] | MS/MS fragments | ScoreMetaboscape | Properties | Reference | Structure |
|---|---|---|---|---|---|---|---|---|---|---|---|
| Maculosin | 1 | 2,72 | 261,123 | C14H16N2 O3 | 261,1234 | -0,8 | 261.1227, 233.1276, 155.0799, 154.0714, 147.0439, 136.0749, 107.0487, 98.0606, 91.0519, 70.0652 | – | Phytotoxic | Vansteelandt et al., Fungal Biology (2012), 116, 954-961 | 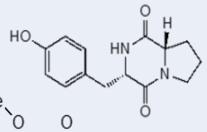 |
| Penicillic Acid | 2 | 4,51 | 171,065 | C8H10O4 | 171,0652 | 0,0 | 125.0595, 97.0649, 69.0338, 43.0180 | 967 | Phytotoxic, Antibiotic | Reiss, J., Deutsche Lebensmittel-Rundschau (1988), 84, 318-20 | 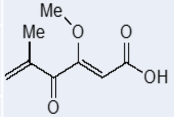 |
| Cyclo (Leu-Pro) | 3 | 4,58 | 211,144 | C11H18N2 O2 | 211,1439 | 0,0 | 183.1493, 155.1535, 138.1275, 114.0915, 98.0602, 86.0969, 70.0653 | 888 | Antibiotic | Wang et al., Chemistry of Natural Compounds (2014), 50, 405-407 | 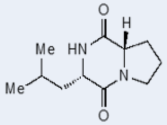 |
| Cyclo (Phe-Pro) | 4 | 5,44 | 245,1285 | C14H16N2 O2 | 245,1285 | 0,0 | 245.1280, 217.1337, 172.1123, 154.0739, 153.0661, 120.0808, 103.0542, 98.0602, 70.0654 | – | Antibiotic | Ai et al., Ann Microbiol (2019) 69:1247–1257 | 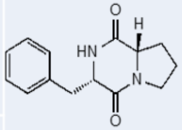 |
| Aurantiamine | 5 | 6,86 | 303,1814 | C16H22N4 O2 | 303,1816 | -0,7 | 275.1856, 260.1258, 245.1211, 235.1191, 217.0720, 204.1124, 192.0635, 176.1179, 158.1088, 149.1072, 147.1001, 133.0756, 122.0964 | 937 | Cell Cycle Inhibitor | Larsen et al., Phytochemistry (1992), 31, 1613-15 | 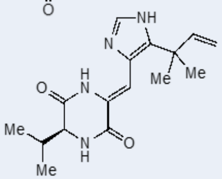 |
| Patulin | 6 | 8,37 | 155,0338 | C7H6O4 | 155,0339 | -0,6 | 53.038, 43.0183 | 612 | Phytotoxic, Cytotoxic | Vansteelandt et al., Fungal Biology (2012), 116, 954-961 | 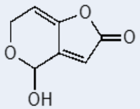 |
| Auranthine | 7 | 8,62 | 331,1189 | C19H14N4 O2 | 331,1190 | -0,3 | 249.0661, 221.0719, 193.0759, 146.0236, 130.0294, 83.0603, 56.0498 | 842 | Antibiotic | Kalinina et al., J. Nat. Prod. (2018), 81, 2177-2186 | 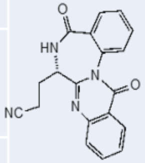 |
| Verrucosidin | 8 | 9,18 | 417,2266 | C24H32O6 | 417,2272 | -1,4 | 399.2157, 345.1689, 317.1752, 249.1113, 221.1176, 197.0806, 181.0852, 153.0547, 133.1010, 97.0649, 43.0180 | – | Neurotoxic | Ganguli et al., Journal of Organic Chemistry (1984), 49(20), 3762-6 | 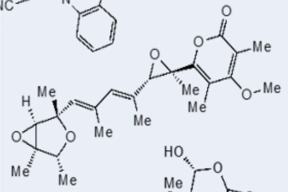 |
| Fuegin | 9 | 9,42 | 267,1591 | C15H22O4 | 267,1591 | 0,0 | 155.0460, 116.9942, 98.9847, 57.0700 | 895 | assumed cytotoxic | Sultana et al., Planta Med (2011), 77, 1848–1851 | 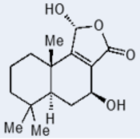 |

**Table S3** Calculated correlations between examined variables and PC1 and PC2

| | PC1 | PC2 | PC3 | PC4 | PC5 | PC6 | PC7 | PC8 |
|---|---|---|---|---|---|---|---|---|
| t0 | 0.29 | 0.75 | 0.57 | 0.19 | 0.01 | 0 | 0 | 0 |
| X1d | 0.96 | 0.1 | 0.06 | -0.23 | 0.06 | 0.06 | 0.04 | -0.01 |
| X7d | 0.98 | 0.04 | 0.04 | -0.16 | 0.05 | 0 | -0.05 | 0.03 |
| X14d | 0.99 | -0.01 | 0 | -0.03 | -0.11 | 0.04 | -0.03 | -0.02 |
| X21d | 0.99 | -0.07 | -0.05 | 0.05 | -0.11 | -0.04 | 0.04 | 0.02 |
| X28d | 0.99 | -0.05 | -0.03 | -0.05 | 0.06 | -0.09 | 0 | -0.03 |
| X63d | 0.83 | -0.32 | -0.15 | 0.42 | 0.06 | 0.03 | 0 | 0 |
| untreat | -0.07 | -0.61 | 0.79 | -0.03 | 0 | 0 | 0 | 0 |

### *Test for the Presence of Viral Plant Pathogens in the Mycelium of P. aurantiogriseum*

A test for viral plant pathogens in the mycelium of *Penicillium aurantiogriseum* was a prerequisite for using the mycelia for plant growth studies. For this purpose, the fungus was cultivated on two different agars (Czapek and Sabouraud). Three samples of the grey mycelium/spore masses from each agar plate were taken with a spatula and transferred into 1.5 ml Eppendorf tubes. 1 ml of 0.1 M potassium phosphate buffer pH 7 was added. The tubes were vigorously vortexed for 30 sec. 50 µl of each suspension were pipetted onto a laboratory film. Pioloform coated grids were placed with the filmed side down onto the suspension drops and left for ca. 5 minutes. Coated sides were rinsed with distilled water containing 100 µM Bacitracin as wetting agent. After dipping the grids vertically onto paper, the grids were stained by touching the filmed grid side on a drop of 2% uranyl-acetate in distilled water, followed by drying the grids, filmed side upwards. After drying, the grids were transferred into a Zeiss 109 electron microscope and checked under a 50 KV electron beam at 108,000-fold magnification for virus-like particles. According to the check, common plant viral pathogens that could be responsible for the death of *Camelina* seedlings were not present in the mycelium.
